# Coalescent-based delimitation outperforms distance-based methods for delineating less divergent species: the case of *Kurixalus odontotarsus* species group

**DOI:** 10.1038/s41598-017-16309-1

**Published:** 2017-11-23

**Authors:** Guohua Yu, Dingqi Rao, Masafumi Matsui, Junxing Yang

**Affiliations:** 1 State Key Laboratory of Genetic Resources and Evolution, Kunming Institute of Zoology, Chinese Academy of Sciences, 32 Jiaochang Donglu, Kunming, Yunnan 650223 China; 2 Graduate School of Human and Environmental Studies, Kyoto University, Yoshida Nihonmatsu, Kakyo-ku, Kyoto, 606-8501 Japan

## Abstract

Few empirical studies have compared coalescent-based methods to distance-based methods for delimitation of less divergent species. In this study, we used two coalescent-based (BFD and BPP) and two distance-based barcoding (ABGD and jMOTU) methods to delimit closely related species in the *Kurixalus odontotarsus* species group. Phylogenetic analyses revealed that the *K. odontotarsus* species group comprises 11 distinct maternal clades with strong support values. Based on the genetic and morphological evidences, we consider that species diversity in the *K. odontotarsus* species group was underestimated and the 11 clades represent 11 species, of which six are unnamed. The coalescent-based delimitations decisively supported the scenario of 11-species corresponding to the 11 clades. However, the distance-based ABGD only obtained 3–6 candidate species, which is not consistent with morphological evidence. These results indicate that BFD and BPP are more conservative than ABGD to false negatives (lumping). Method of fixed threshold (jMOTU) may obtain a resolution similar to that inferred by BFD and BPP, but it severely relies on subjective choice of the threshold and lacks statistical support. We consider that coalescent-based BFD and BPP approaches outperform distance-based methods for delineation of less divergent species.

## Introduction

Species, which is viewed as the currency of biology^1^, are the fundamental units in most subdisciplines of biology^2^, and they play a central role in systematic studies and comparative analyses in ecology, evolution, conservation and biogeography^3^. As such, robust measures of species delimitation and boundaries are crucial to understanding the evolution of organisms and how best to manage biodiversity in the face of increased anthropogenic pressure^4^.

DNA barcoding based on a short fragment of DNA (typically mitochondrial COI sequences) has attracted attention with promises to aid in species identification and discovery and has been widely used for species delimitation. This method requires subjective decisions regarding the thresholds (gap between intraspecific and interspecific divergences) that mark the species boundary^5^ and so it had been argued for a long time (e.g.,^6–9^). Besides false positives (splitting; identification of spurious novel taxa), another serious problem of distance-based DNA barcoding is that its bias against delineating closely related young species (lumping; false negatives), as this will lead to an underestimation of species diversity^6,8,10–12^.

Species discovery should be amenable to statistical exploration^13,14^ and during recent years some coalescent-based statistical methods for species delimitation have been developed (e.g.,^15–17^). Coalescent-based species delimitation methods use probabilistic approaches that do not require reciprocal monophyly of alleles or fixed differences, which is not expected for most alleles, particularly at the timescale of recent speciation^18^. By applying probabilistic models, these methods provide clear and objective testing of alternative hypotheses of evolutionary independence and will improve the discovery, resolution, consistency, and stability of the taxonomy of species^19^. However, it has been suggested that coalescent-based species delimitation methods may also erroneously lump recently evolved species^19,20^. Although some attempts have been conducted to compare the performances of different coalescent-based methods (e.g.,^17,21^), few empirical studies have compared these methods with distance-based DNA barcoding on the delimitation of less divergent species.

**Figure 1:**
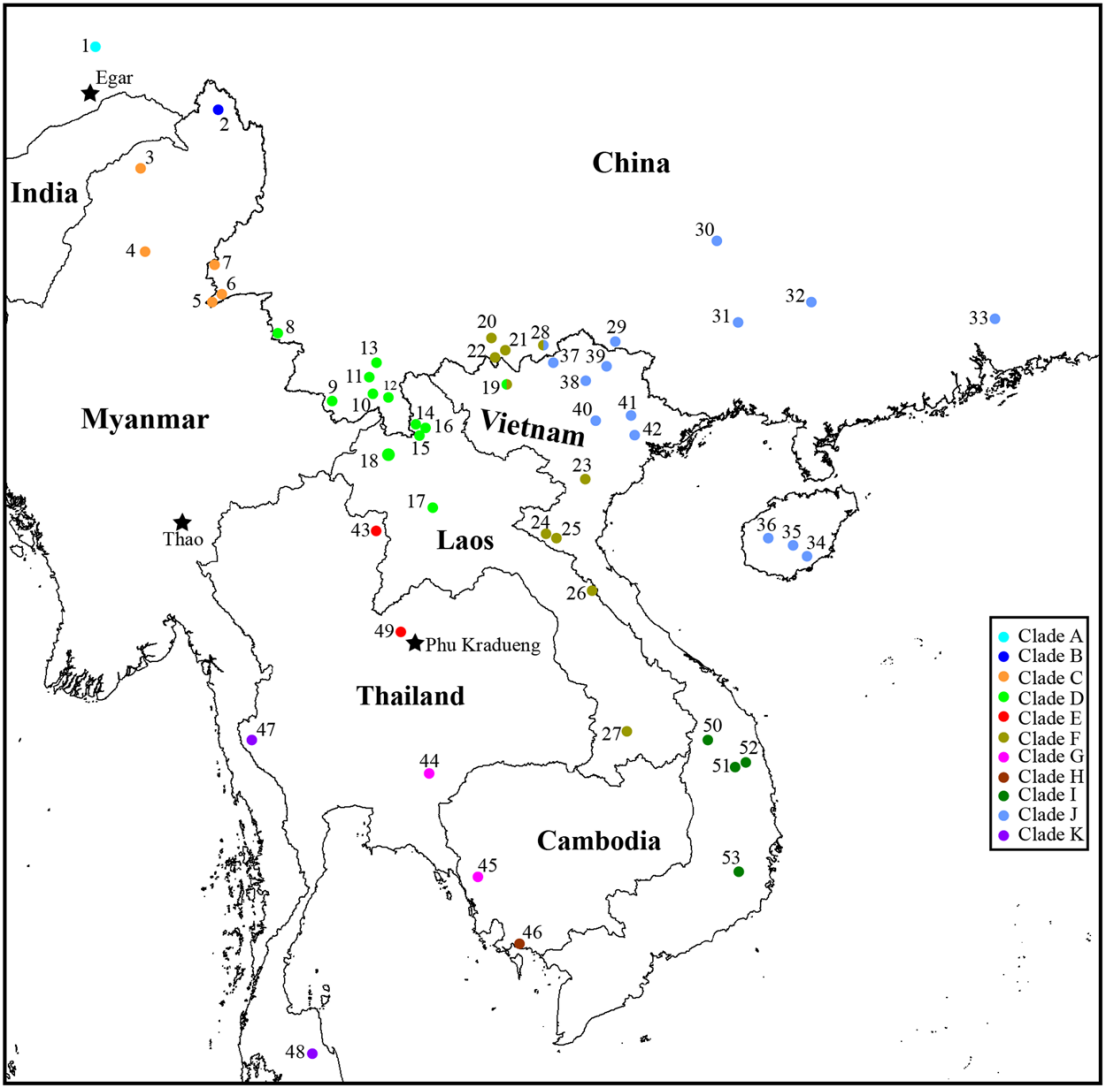
Collection sites for the samples of the *K. odontotarsus* species group used in this study. Sites are numbered as in Table S1 and clades are highlighted by different color. The base map was created in DIVA-GIS v.7.5.0 (http://www.diva-gis.org) and then edited in Illustrator CS6 v.16.0 (http://www.adobe.com/products/illustrator.html).

Here, we investigate the performances of two coalescent-based (Bayes factor delimitation [BFD]^17^ and Bayesian phylogenetics and phylogeography [BPP]^16^) and two distance-based DNA barcoding (Automated Barcode Gap Discovery [ABGD]^22^ and jMOTU^23^) methods on delineating less divergent members of the *Kurixalus odontotarsus* species group alongside available morphological evidence. The *K. odontotarsus* species group distributes wildly in Himalayan front ranges (Fig. 1), and currently five species (*K. odontotarsus*, *Kurixalus bisacculus*, *Kurixalus naso*, *Kurixalus baliogaster*, and *Kurixalus verrucosus*) are recognized in it^24^. Owing to the morphological conservation and lack of a thorough taxonomic study, species boundary in this group is still very obscure. Inger *et al*.^25^ considered that the Chinese and Vietnamese records of *K. odontotarsus* probably represent *K. verrucosus* or *K. bisacculus*, Orlov *et al*.^26^ also considered Vietnamese *K. odontotarsus* as *K. verrucosus*, and *Kurixalus hainanus* was thought to be a synonym of *K. odontotarsus* by some authors (e.g.,^27,28^). Yu *et al*.^29^ proposed that *K. odontotarsus*, *K. bisacculus*, and *K. verrucosus* should be treated as three independent species and suggested placing *K. odontotarsus* from Tibet and *K. hainanus* in *K. verrucosus* and *K. bisacculus*, respectively. However, this earlier work only involved limited samples and lacked statistical test for the species reassignments, which maybe leads to an incomprehensive understanding on the species boundary among *K. odontotarsus* species group.

## Results

### Sequence characteristics, mtDNA phylogeny, and nuDNA network

The length of COI sequences in dataset I was 807 bp, which included 274 variable sites and 229 parsimony informative sites. For the dataset II, alignments of 12S rRNA and 16S rRNA genes yielded 401 and 875 sites, respectively; of the 1276 positions, 405 were variable and 276 were parsimony informative. For dataset III, length of 12S rRNA, 16S rRNA, and COI alignments was 401 bp, 868 bp, and 807 bp, respectively. Saturation was not observed for 12S rRNA, 16S rRNA, and COI genes (Supplementary Fig. S1). The length of Tyr, BDNF, and Rag-1 genes was 521 bp, 701 bp, and 926 bp, respectively.

**Figure 2:**
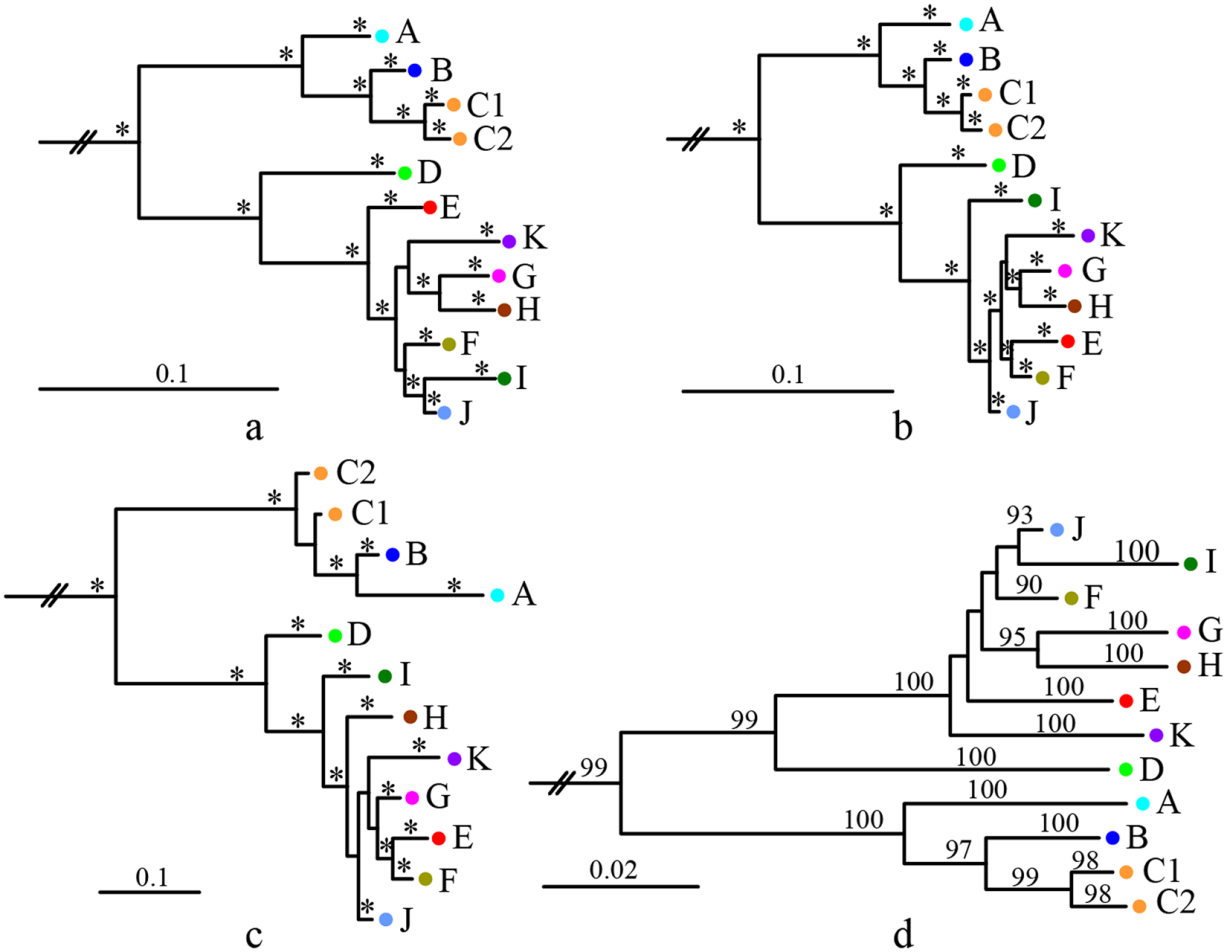
Simplified Bayesian (**a**–**c**) and Neighbor-joining (**d**) dendrograms. a, COI; b, COI and rRNA; c, rRNA; d, COI; *posterior probabilities >95%. The complete trees are presented in Supplementary Figs S2–S5.

All Bayesian and NJ analyses concordantly identified 11 distinct clades (labeled A–K) in the *K. odontotarsus* species group with high support values except for Bayesian analysis of rRNA sequences (Fig. 2), and distributions of these clades do not overlap obviously (Fig. 1). The average uncorrected p-distances of COI sequences between and within these clades range from 3.35% to 13.85% and from 0 to 1.35%, respectively (Table 1).

**Table 1:** The average p-distance between clades estimated from COI sequences.

Clade	**I** (0.5%)	**D** (0.23%)	**F** (1.00%)	**J** (1.35%)	**E** (0.06%)	**G** (0)	**K** (0.74%)	**H** (0)	**B** (0.17%)	**C** (0.52%)	**A** (0.05%)
**I**	—										
**D**	9.21%	—									
**F**	4.48%	9.10%	—								
**J**	3.73%	9.02%	3.50%	—							
**E**	4.84%	8.97%	4.01%	3.94%	—						
**G**	5.45%	10.19%	4.52%	4.18%	4.80%	—					
**K**	5.74%	10.12%	5.28%	5.09%	5.24%	5.33%	—				
**H**	5.49%	9.14%	4.83%	4.18%	4.80%	3.35%	5.20%	—			
**B**	12.60%	12.72%	12.50%	13.02%	11.80%	12.89%	12.50%	13.26%	—		
**C**	13.34%	12.93%	12.96%	13.21%	12.78%	13.38%	13.20%	13.68%	3.51%	—	
**A**	12.86%	12.60%	13.19%	13.15%	13.14%	13.60%	12.92%	13.85%	5.19%	6.18%	—

Values in the parentheses of the first row are COI p-distance within clade.

**Figure 3:**
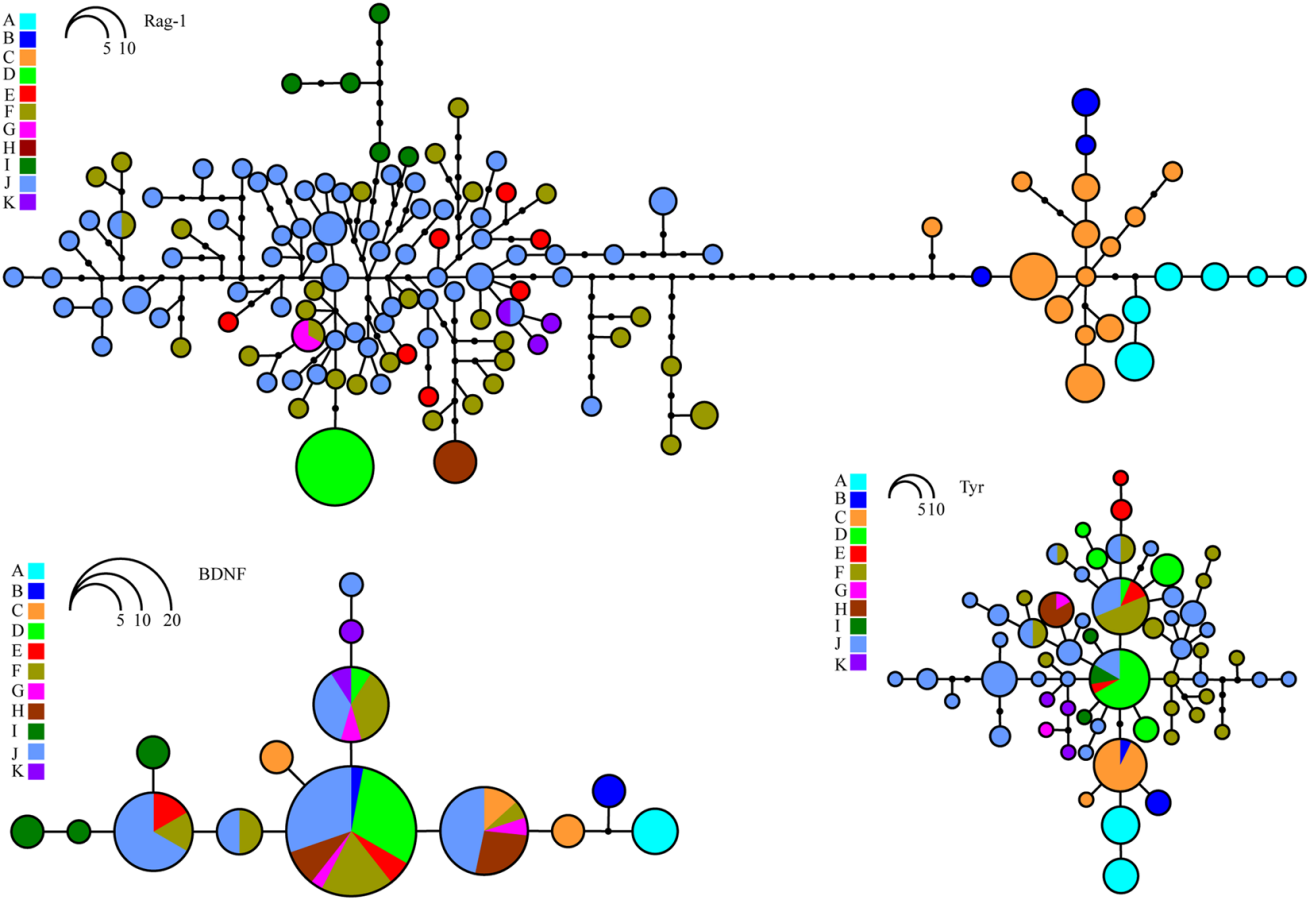
Haplotype network inferred from the three nuclear loci.

Among the three nuclear loci, Rag-1 got a higher level of resolution of haplotype network than Tyr and BDNF (Fig. 3). For example, the Rag-1 network indicated that *K. odontotarsus* (clade D) and clade H do not share haplotype with other clades, whereas both Tyr and BDNF networks showed haplotype sharing between these two clades. Although relationships among most clades were not resolved by the nuclear genes, network analyses of all the three nuclear loci indicated that individuals from Tibet do not share alleles with individuals from other places and they form a distinct clade (clade A).

### Coalescent-based Species delimitation

On the basis of the clades obtained by phylogenetic analyses of mtDNA sequences, nine species delimitation scenarios were tested by BFD approach based on the rRNA (12S and 16S rRNA) and COI data, respectively (Table 2). For the rRNA data, the model of four-species being consistent with current taxonomy got the lowest estimations of both path sampling (PS)^30^ and stepping-stone (SS)^31^ values; by splitting the species group into more species, PS and SS values increased (with the exception of the split of C1 and C2) and the model of 11-species corresponding to the 11 clades received decisive support over all other models (2*ln*Bf = 10.82–208.5 and 10.52–208.22 in PS and SS analyses, respectively; Table 2). BFD analyses based on COI sequences obtained a completely same pattern and the scenario of 11-species was decisively superior to the hypotheses of 4–10 species (2*ln*Bf = 29.42–218.88 and 29.22–218.22 in PS and SS analyses, respectively; Table 2).

**Table 2:** Marginal likelihood and Bayes factor estimation for different species delimitation models.

Species delimitation model	MLE (PS)	2*ln*Bf (PS)	MLE (SS)	2*ln*Bf (SS)
**Based on 12S and 16S rRNA sequence**s				
1) 4 species: (ABC) (D) (I) (EFGHJK)	−6693.23	208.5	−6693.10	208.22
2) 5 species: (BC) (D) (I) (EFGHJK) (A)	−6677.85	177.74	−6677.55	177.12
3) 6 species: (C) (D) (I) (EFGHJK) (A) (B)	−6674.92	171.88	−6674.80	171.62
4) 7 species: (C1) (D) (I) (EFGHJK) (A) (B) (C2)	−6675.46	172.96	−6675.59	173.2
5) 7 species: (C) (D) (I) (FGHJK) (A) (B) (E)	−6666.62	155.28	−6666.77	155.56
6) 8 species: (C) (D) (I) (GHJK) (A) (B) (E) (F)	−6611.97	45.98	−6611.80	45.62
7) 9 species: (C) (D) (I) (HJK) (A) (B) (E) (F) (G)	−6606.37	34.78	−6606.51	35.04
8) 10 species: (C) (D) (I) (JK) (A) (B) (E) (F) (G) (H)	−6594.39	10.82	−6594.25	10.52
9) 11 species: (C) (D) (I) (K) (A) (B) (E) (F) (G) (H) (J)	−**6588.98**	—	−**6588.99**	—
**Based on COI sequences**				
1) 4 species: (ABC) (D) (I) (EFGHJK)	−4587.19	218.88	−4586.91	218.22
2) 5 species: (BC) (D) (I) (EFGHJK) (A)	−4572.54	189.58	−4572.34	189.08
3) 6 species: (C) (D) (I) (EFGHJK) (A) (B)	−4568.36	181.22	−4568.32	181.04
4) 7 species: (C1) (D) (I) (EFGHJK) (A) (B) (C2)	−4568.85	182.2	−4568.25	180.9
5) 7 species: (C) (D) (I) (FGHJK) (A) (B) (E)	−4557.51	159.52	−4557.41	159.22
6) 8 species: (C) (D) (I) (GHJK) (A) (B) (E) (F)	−4512.49	69.48	−4512.35	69.10
7) 9 species: (C) (D) (I) (HJK) (A) (B) (E) (F) (G)	−4510.36	65.22	−4510.10	64.60
8) 10 species: (C) (D) (I) (JK) (A) (B) (E) (F) (G) (H)	−4492.46	29.42	−4492.41	29.22
9) 11 species: (C) (D) (I) (K) (A) (B) (E) (F) (G) (H) (J)	−**4477.75**	—	−**4477.80**	—

Clades obtained by phylogenetic analysis (A–K) were assigned to different species.

Consistent with BFD approach, when the clade C was treated as a single species, all analyses of BPP method also supported the existence of 11 species that correspond to the 11 clades (A–J) with posterior probability greater than 98%, and the posterior probability of each delimited species was also greater than 98% (Table 3). However, when the clade C was split into two separate species (C1 and C2), the model of 12-species was also supported with high posterior probability (Table 3).

**Table 3:** Results of unguided Bayesian species delimitation (BPP) based on the combination of rRNA and COI sequences.

Delimited species	Posterior											
	*θ*~G (2, 50), τ~G (2, 50)				*θ*~G (2, 1000), τ*~*G (2, 1000)				*θ~*G (2, 50), τ*~*G (2, 1000)			
	A0		A1		A0		A1		A0		A1	
	S = −1	S = −145	S = −1	S = −145	S = −1	S = −145	S = −1	S = −145	S = −1	S = −145	S = −1	S = −145
**Model 1: treat lineage C as a single species**												
Species number = 11	0.9914	0.9924	0.9895	0.9909	1.0000	1.0000	1.0000	1.0000	0.9936	0.9937	0.9932	0.9928
A	1.0000	1.0000	1.0000	1.0000	1.0000	1.0000	1.0000	1.0000	1.0000	1.0000	1.0000	1.0000
B	0.9999	1.0000	1.0000	0.9999	1.0000	1.0000	1.0000	1.0000	0.9998	0.9993	0.9986	0.9987
C	1.0000	1.0000	1.0000	1.0000	1.0000	1.0000	1.0000	1.0000	1.0000	1.0000	1.0000	1.0000
D	1.0000	1.0000	1.0000	1.0000	1.0000	1.0000	1.0000	1.0000	1.0000	1.0000	1.0000	1.0000
E	0.9992	0.9999	0.9996	0.9995	1.0000	1.0000	1.0000	1.0000	0.9996	0.9990	0.9995	0.9996
F	1.0000	1.0000	1.0000	1.0000	1.0000	1.0000	1.0000	1.0000	1.0000	1.0000	1.0000	1.0000
G	0.9918	0.9926	0.9899	0.9910	1.0000	1.0000	1.0000	1.0000	0.9938	0.9944	0.9942	0.9939
H	1.0000	1.0000	1.0000	1.0000	1.0000	1.0000	1.0000	1.0000	1.0000	1.0000	1.0000	1.0000
I	0.9996	0.9998	0.9994	0.9997	1.0000	1.0000	1.0000	1.0000	0.9998	0.9996	0.9995	0.9991
J	1.0000	1.0000	1.0000	1.0000	1.0000	1.0000	1.0000	1.0000	1.0000	1.0000	1.0000	1.0000
K	0.9922	0.9925	0.9901	0.9917	1.0000	1.0000	1.0000	1.0000	0.9941	0.9951	0.9946	0.9942
**Model 2: split lineage C into two species (C1 and C2)**												
Species number = 12	0.9749	0.9812	0.9798	0.9807	1.0000	1.0000	1.0000	1.0000	0.9870	0.9854	0.9841	0.9884
A	1.0000	1.0000	1.0000	1.0000	1.0000	1.0000	1.0000	1.0000	1.0000	0.9999	1.0000	1.0000
B	0.9840	0.9903	0.9895	0.9905	1.0000	1.0000	1.0000	1.0000	0.9959	0.9950	0.9944	0.9978
C1	0.9839	0.9901	0.9895	0.9903	0.9999	1.0000	1.0000	1.0000	0.9946	0.9929	0.9918	0.9944
C2	0.9999	0.9998	0.9999	0.9999	0.9999	1.0000	1.0000	1.0000	0.9999	0.9999	0.9999	0.9999
D	1.0000	1.0000	1.0000	1.0000	1.0000	1.0000	1.0000	1.0000	1.0000	1.0000	1.0000	1.0000
E	0.9997	0.9997	0.9994	0.9991	1.0000	1.0000	1.0000	1.0000	0.9945	0.9996	0.9994	0.9994
F	1.0000	1.0000	1.0000	1.0000	1.0000	1.0000	1.0000	1.0000	0.9999	1.0000	1.0000	1.0000
G	0.9906	0.9912	0.9905	0.9907	1.0000	1.0000	1.0000	1.0000	0.9913	0.9918	0.9912	0.9928
H	1.0000	1.0000	1.0000	1.0000	1.0000	1.0000	1.0000	1.0000	1.0000	1.0000	0.9999	0.9999
I	0.9997	0.9996	0.9999	0.9997	1.0000	1.0000	1.0000	1.0000	0.9996	0.9991	0.9988	0.9992
J	1.0000	1.0000	1.0000	1.0000	1.0000	1.0000	1.0000	1.0000	1.0000	1.0000	1.0000	1.0000
K	0.9908	0.9916	0.9908	0.9913	1.0000	1.0000	1.0000	1.0000	0.9932	0.9923	0.9929	0.9932

A0, algorithm 0; A1, algorithm 1; S, seed.

### Distance-based barcoding for COI

**Figure 4:**
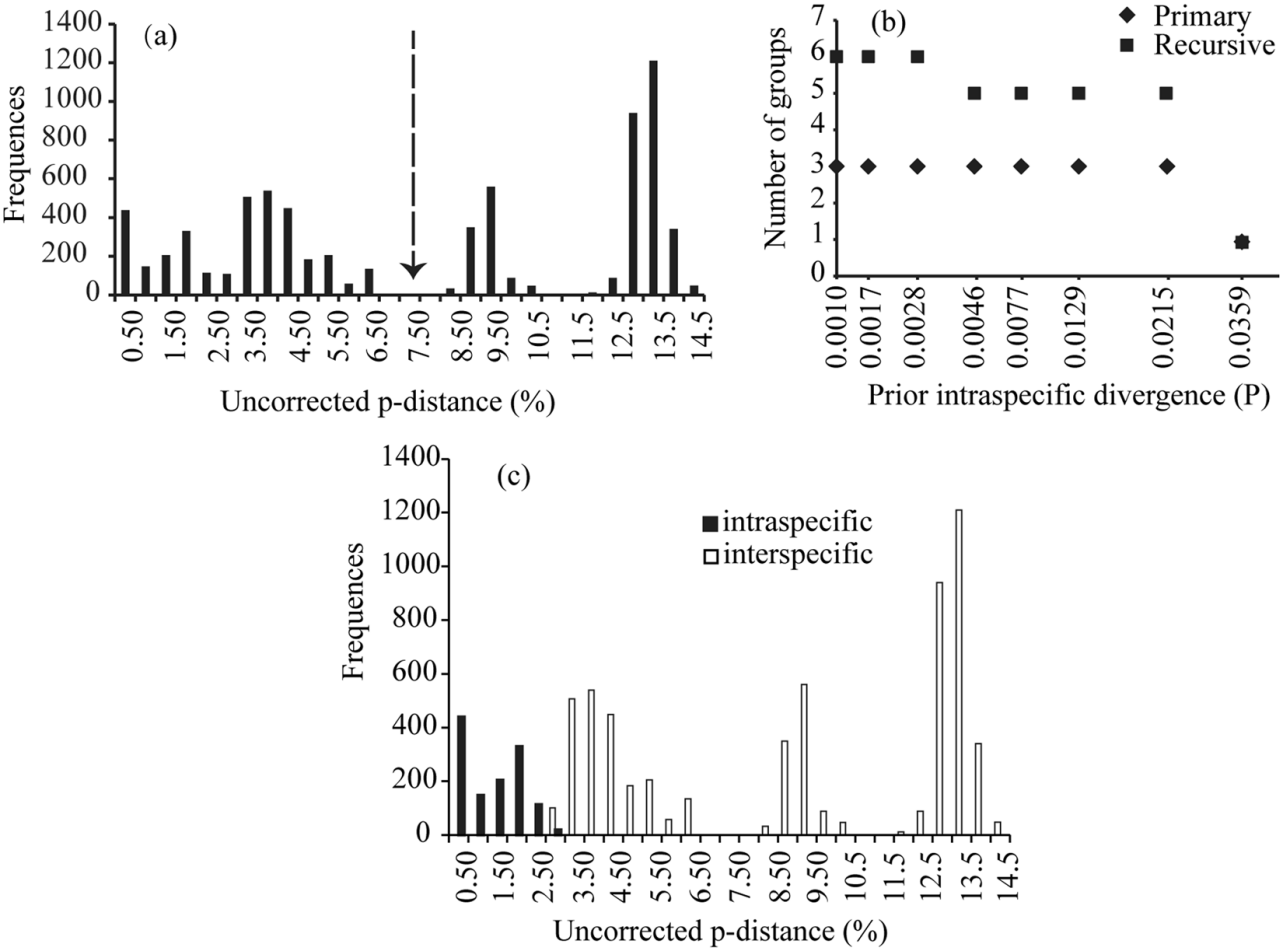
(**a**) Distribution of pairwise distance of COI; (**b**) ABGD partition obtained from COI; (**c**) distribution of intraspecific and interspecific divergences of COI.

Distribution of pairwise distances showed an “observed” divergence gap for COI sequences (*ca*. 7.5%; Fig. 4a). The primary analysis of ABGD obtained three primary groups for all prior *P* (Fig. 4b); one group includes clades A, B, and C, one includes clade D, and one consists of clades E–K. The recursive analysis recovered 6 recursive groups for prior *P* from 0.1% to 0.28% ([A], [B], [C1], [C2], [D], [E–K]) and 5 recursive groups for prior *P* from 0.46% to 2.15% ([A], [B], [C], [D], [E–K]).

**Figure 5:**
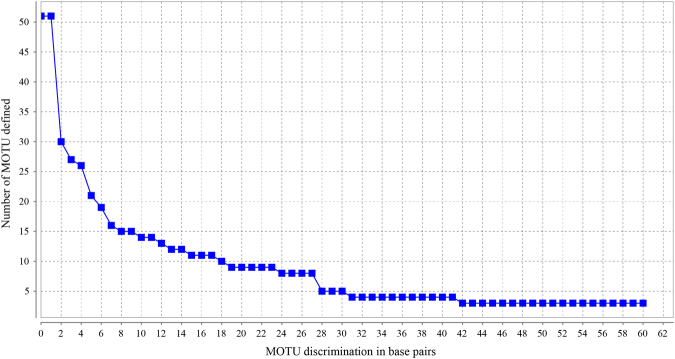
Chart showing number of MOTU defined at each cutoff value from 1 to 60 bp.

The jMOTU analysis detected 51–3 molecular operational taxonomic units (MOTU) with the increase of threshold value from 1 to 60 bp (Fig. 5) and 11 MOTU that correspond to the 11 clades were recognized when the threshold value was 15, 16 or 17 bp (corresponding to 1.89%, 2.02% or 2.14% of mean length of the sequences, respectively). When the threshold value was set to 42–60 bp (corresponding to 5.29%–7.56% of mean length of the sequences), only three MOTU were identified; one comprises of clades A–C, one comprises of clade D, and one comprises of clades E–K.

## Discussion

Hitherto, no study based on a wide sampling has been done to investigate the species diversity among the species group of *K. odontotarsus*, although there were some disputes on the taxonomy and species boundary among this group^25–29^. In this study, on the basis of a wide sampling, our phylogenetic analyses strongly supported the existence of 11 mitochondrial clades (A–K) in this group (Fig. 2) and distributions of these clades do not overlap obviously (Fig. 1), although most of these genealogies were not distinguished by the three nuclear protein-coding loci (Fig. 3) probably owing to slow rates of mutation and/or incomplete lineage sorting of these nuclear genes. Alongside available morphological evidence, we think that the species diversity of *K. odontotarsus* species group was underestimated in the past and some taxonomic changes need to be made.

**Figure 6:**
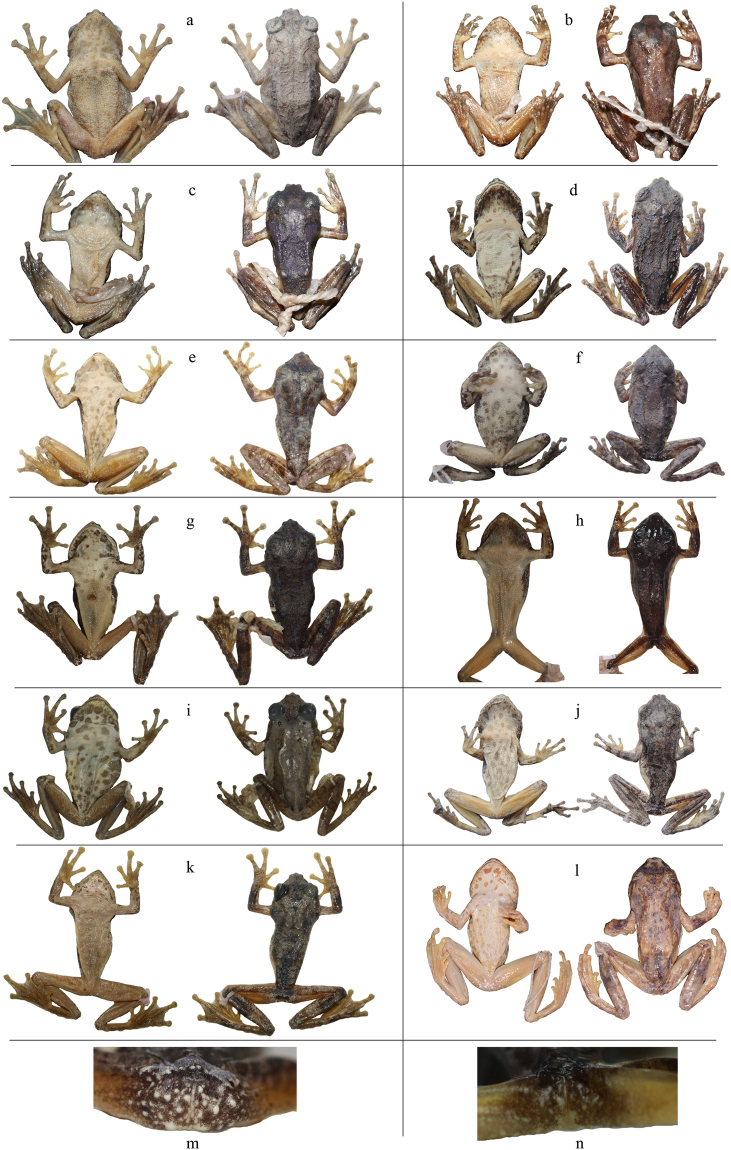
(**a**) Ventral and dorsal views of Rao 06301 in clade A; (**b**) ventral and dorsal views of CAS 224563 in clade B; (**c**) ventral and dorsal views of CAS 231491 in clade C; (**d**) ventral and dorsal views of YGH 090131 in clade D; (**e**) ventral and dorsal views of KUHE 19333 in clade E; (**f**) ventral and dorsal views of 1506229 in clade F; (**g**) ventral and dorsal views of FMNH 265820 in clade G; (**h**) ventral and dorsal views of FMNH 261900 in clade H; (**i**) ventral and dorsal views of *K. baliogaster* (FMNH 252839); (**j**) ventral and dorsal views of Rao 14111302 in clade J; (**k**) ventral and dorsal views of KUHE 35069 in clade K; (**l**) ventral and dorsal views of the holotype of *K. verrucosus* (BMNH 1893.10.9.23); (**m**) vent of FMNH 265820 in clade G; (**n**) vent of FMNH 261900 in clade H. The images in b and c were taken by Jens V. Vindum, images in g, h, i, m, and n were taken by Rachel Grill, and images in l were taken by Jeff Streicher.

Population from Motuo, China originally was recognized as *K. odontotarsus* ^27,28^ and then was tentatively placed in *K. verrucosus* by Yu *et al*.^29^ because genetically it was clustered together with “*K. verrucosus*” from Kachin, Myanmar. This taxonomic revision was followed by subsequent molecular studies^32–34^, but no morphological comparison has been done to justify it. In this study, both mitochondrial and nuclear evidences revealed that specimens from Motuo form a distinct clade (labeled A), indicating that this clade represents an independent evolutionary lineage. After checking the holotype of *K. verrucosus*, we found that female specimens from Motuo obviously differ from *K. verrucosus* by having a dermal appendage on the pointed snout and finely granular chin and chest (versus snout rounded with no dermal appendage and chin and chest smooth in *K. verrucosus* ^35^) (Fig. 6; Supplementary Table S1), indicating that clade A is actually not *K. verrucosus*. *Kurixalus naso* was described by Annandale^36^ based on a female specimen from southern Tibet^37^ and it is characteristic of the dermal appendage on its pointed snout^36^. Geographically our collection site of specimens in clade A is close to the type locality of *K. naso* (Fig. 1) and morphologically the female from Motuo, Tibet agrees well with the holotype of *K. naso* by having a dermal appendage on the snout and granular ventral surface. So we think that clade A actually represents *K. naso*.

The five specimens from Kachin, Myanmar in clade B and sub-clade C1 were treated as *K. verrucosus* in Yu *et al*.^29^. However, clades B and C have finely granular chin and breast and pointed snout with dermal appendage, whereas snout of *K. verrucosus* is rounded with no dermal appendage and the chin and breast of *K. verrucosus* are smooth^35^ (Fig. 6; Supplementary Table S1). Thus, neither of these two clades belongs to *K. verrucosus*. Additionally, clade B differs from clade C by having a smaller body size, more dark spots on venter, and numerous small white warts on dorsal surface (Fig. 6; Supplementary Table S1). Therefore, we consider that they represent two unnamed lineages pending further morphological study.

Clade D refers to *K. odontotarsus*, which was considered to distribute widely in southern China^24,27,28^. Yu *et al*.^29^ revealed that distribution of *K. odontotarsus* in China should be limited to its type locality and nearby regions. Based on the present study, we think that *K. odontotarsus* only distributes in Yunnan, northern Laos, northern Vietnam, and probably eastern Myanmar (Fig. 1). Clade I represents *K. baliogaster*, which obviously differs from other members of the species group by lacking dermal flaps or fringes on the limbs and having smooth dorsal and lateral skin^25^ (Fig. 6; Supplementary Table S1). Clade E is consisted of specimens from two localities of northern Thailand (Pua, Nan and Phu Luanag, Loei). Morphologically specimens in clade E agree with *K. bisacculus* in having paired external lateral vocal sacs^38^ and geographically Phu Luanag is very close to the type locality of *K. bisacculus* (Fig. 1). So we consider that clade E represents *K. bisacculus* sensu stricto.

*Kurixalus hainanus* was originally placed in *K. odontotarsus* ^26^ and later was described as a new species by Zhao *et al*.^39^. Yu *et al*.^29^ considered *K. hainanus* synonym of *K. bisacculus*, which was followed by most studies^33,34,40^ with the exception of Li *et al*.^32^. In the present study, all specimens from Hainan Island were grouped in clade J. So here we refer to clade J as *K. hainanus*. Genetically the divergence between *K. hainanus* (clade J) and *K. bisacculus* (clade E) is 3.94%, which is greater than the divergence between *K. hainanus* and *K. baliogaster* (3.73%) (Table 1), and morphologically *K. hainanus* differs from *K. bisacculus* by having single internal vocal sac^39^ and less pointed snout (Fig. 6; Supplementary Table S1). Therefore, we admit that *K. hainanus* is a valid species rather than synonymy of *K. bisacculus*. Additionally, *Kurixalus hainanus* can be distinguished from *K. odontotarsus* by having forked omosternum^29,39^ and less pointed snout and from *K. verrucosus* by having granular throat and chest (versus smooth; Fig. 6).

Clade F was also placed in *K. bisacculus* by Yu *et al*.^29^ based on molecular evidence from 12 S and 16 S rRNA. In this study, this clade was recovered as the sister to *K. bisacculus* (clade E) or the sister to the clade of *K. baliogaster* (clade I) and *K. hainanus* (clade J) (Fig. 2). The divergence between clade F and *K. bisacculus* is 4.01%, which is slightly greater than the divergence between *K. bisacculus* and *K. hainanus* (3.94%; Table 1). Morphologically clade F differs from *K. bisacculus* by having an internal subgular vocal sac and bigger body size (Supplementary Table S1); from *K. baliogaster* (clade I) by having coarse dorsal surface and dermal flaps or fringes on the limbs; and from *K. hainanus* (clade J) and *K. verrucosus* by having more pointed snout (Fig. 6; Supplementary Table S1). So we think that clade F represents an unnamed lineage.

Specimens in clade H were identified as *K. bisacculus* by Stuart & Emmett^41^, but Li *et al*.^32^ treated FMNH 261900 as a unnamed species with no discussion. Here we found that the divergence between this clade and *K. bisacculus* (clade E) is 4.80%, which is greater than some inter-specific divergences between other species (Table 1). Clade H differs from *K. bisacculus* by having few or no dark spots on venter (Fig. 6). Moreover, clade H also differs from *K. verrucosus* by having pointed snout and granulated chin and breast^35^ and from *K. odontotarsus* (clade D), *K. baliogaster* (clade I), clade F, and *K. hainanus* (clade J) by having few or no dark spots on the belly and smaller body size^41^ (Fig. 6; Supplementary Table S1). So we think that clade H represents an unnamed species.

Clade G is the sister to clade H and the divergence between them is 3.35%. Morphologically clade G is different from clade H in that the former possesses more prominent white tubercles around the vent and its chin is scattered with black spots, whereas the later has less white tubercles and its chin is clouded brownish (Fig. 6). Moreover, clade G also differs from *K. verrucosus* by having pointed snouts and granulated chins and breasts (Fig. 6; Supplementary Table S1). So we think that clade G also represents an unnamed lineage.

Clade K was treated as *K. bisacculus* in Nguyen *et al*.^33^. However, genetically the divergence between this clade and *K. bisacculus* (clade E) is 5.24%, which is even greater than the divergence between *K. bisacculus* and *K. baliogaster* (4.84%), and morphologically this clade differs from *K. bisacculus* in that its chin is scattered with black spots, whereas chin of the later is clouded brownish (Fig. 6; Supplementary Table S1). Additionally, clade K also differs from *K. verrucosus* by having pointed snouts and granulated chins and breasts (Fig. 6; Supplementary Table S1). So we think that clade K is not *K. bisacculus* but an unnamed species.

Therefore, species diversity of the *K. odontotarsus* species group seems to be underestimated and six unnamed morphospecies may exist in this group with exceptions of *K. naso*, *K. odontotarsus*, *K. bisacculus*, *K. baliogaster*, *K. hainanus*, and *K. verrucosus*. These taxonomic changes were supported by the coalescent-based Bayes factor delimitation (BFD), which decisively supported the 11 clades as independent species (Table 2). Explicit species delimitation models have the advantage of clarifying more precisely what is being delimited and what assumptions we are making in doing so^42^. A number of benefits exist when using the BFD approach to testing models of species limits^17^. For example, the user does not have to constrain phylogenetic relationships between lineages *a priori*, which can avoid the assumption that the gene trees are inferred without error and the uncertainty of relationship between clades resulted from inadequate phylogenetic signal in the data. It has been suggested that the coalescent-based species delimitation methods may also lump species if species divergence is recent^20^, and results from simulated data in Grummer *et al*.^17^ showed that BFD has the most difficult time in testing species boundaries when investigating the “split” scenario. Obviously this condition did not occur in our empirical data and “splitting” scenarios were strongly supported; the most “splitting” model (11-species corresponding to the 11 mtDNA lineages) received decisive support over other scenarios (Table 2). This should be interpreted as strong signal in the data for these separate lineages as being evolutionarily distinct (species) alongside nonoverlapping distributions (Fig. 1) and abovementioned morphological differences between them. On the other side, coalescent-based delimitation methods may erroneously split populations if there is population subdivision within lineages^14,43^ and incorrect specie delimitation is likely to be inferred when an incomplete sample of a continuously distributed species is analyzed^43^. However, we can exclude this kind of false positive in our BFD analyses because the PS and SS values did not get increase when we split the sub-lineages C1 and C2 into two separate species (Table 2).

Being consistent with the BFD approach, the BPP method also supported the 11 lineages as independent species with high probability. The ancestral population sizes (*θ*) and the root age (τ) can affect the posterior probabilities of tested models^16^ and empirical data indicated that large *θ* should favor fewer species compared with analyses using a smaller *θ* ^44,45^. However, this condition did not occur in some studies (e.g,^46^) and our analyses based on three different combinations of *θ* and τ also produced same species delimitations, indicating that this is not true in this study. Extremely large *θ* and/or small τ mean that the populations may have not evolved into separate species. Probably a better strategy is to set the means of both priors with one order of magnitude based on a preliminary analysis of the data^42^ as we did in this study. Additionally, McKay *et al*.^45^ revealed that BPP is sensitive to the divergent effects of nonrandom mating caused by intraspecific processes such as isolation-with-distance and considered that BPP may not be a conservative method for delimiting independently evolving population lineages, and recently Sukumaran & Knowles^47^ also showed that the multispecies coalescent diagnoses genetic structure, not species and that BPP tends to overestimate the number of true species. We have tested for this concern by splitting the two sub-clades (C1 and C2) of clade C into separate species, and all analyses employing different priors of *θ* and τ also supported them as independent species with high probability (Table 3). Sub-clades C1 and C2 are separated by ~140 km, but for the time being we have no much information to diagnose them as two distinct morphospecies. So, a tentative explanation for the split between C1 and C2 revealed by BPP is that, as McKay *et al*.^45^ pointed out, C1 is likely divergent to some degree from C2 owing to deviation from random mating caused by evolutionary processes such as isolation by distance and BPP is sensitive to it. This is confirmed by the Mantel test, which revealed that isolation by distance was significant in the clade C (r = 0.7539, p = 0.029; Supplementary Fig. S6) although it should be treated with caution because of limited sampling from sites 3 and 4. Thus, external information such as morphological data must be used to correctly attribute the elements of structure delimited by BPP to either species-level or population-level^47^.

As mentioned above, the BFD approach did not decisively support the split of C1 and C2, while BPP analyses also supported them as two independent species with high probability when C1 and C2 were treated as two different species. Similar conditions also occurred in Hotaling *et al*.^48^, in which BPP analyses supported the Tsinjoarivo and Montagned’Ambre populations of mouse lemurs as two lineages whereas BFD analyses did not support them as two species. So, why BFD and BPP produced contradictory results for this scenario? The models implemented in BPP are nested statistical hypotheses where the one-species model is the null model and the two-species model is the alternative model^45^. The null model invokes random mating and possibly any statistically significant departure from random mating will cause statistical methods such as BPP to reject the null model and to support the presence of multiple species^45^. But differing from BPP method, species delimitation models tested by BFD are non-nested^17^, which may make BFD more conservative than BPP to the deviation from random mating caused by evolutionary processes such as isolation by distance.

Previous studies showed that distance-based method ABGD will over-split species into multiple candidate species if deep divergences occur between certain populations (e.g,^49^). In this study, contrary to the two coalescent-based methods, the distance-based barcoding method ABGD revealed that the divergence threshold would be *ca*. 7.5% for COI sequences (Fig. 4a) and there were only three primary groups (candidate species) in the less divergent species group of *K. odontotarsus* (Fig. 4b). Although recursive analysis of ABGD obtained slightly higher estimations of candidate species (five or six), species diversity in the species group were still underestimated by *c*. 50% compared to the coalescent-based delimitations. Moreover, both primary and recursive analyses of ABGD consistently placed clades E–K into a single candidate species (lumping), which is obviously incongruent with the morphological evidences. For instance, *K. baliogaster* (clade I) differs from other members of the species group in the absence of dermal flaps or fringes on the limbs and its smooth dorsal and lateral skin^25^ (Fig. 6).

It has been shown that the gap between interspecific and intraspecific divergences may vary among taxonomic groups or it may not exist at all (e.g.,^50–52^). When we assigned the 11 clades into different species according to the results of coalescent-based delimitations, the interspecific and intraspecific divergences also overlapped slightly (Fig. 4c). Thus, the observed barcode gap in ABGD analysis might be false because it could be produced by grouping young species into a single cluster as illustrated in this case, which will lead to an underestimation of species diversity (false negatives). In other words, ABGD appears to be affected by the mix of deep and shallow divergences^49^ and the results from ABGD could wrongly suggest the presence of a relatively large barcoding gap for less divergent species^53^. Therefore, based on our empirical data, it is suggested that coalescent-based BFD and BPP approaches are more powerful than the distance-based ABGD barcoding approach for delineating less divergent species even with a single locus, being consistent with the simulation of Yang & Rannala^9^, although these two coalescent-based approaches may also lead to false negatives and/or false positives^14,19,20,43,45,47^.

For methods based on a fixed divergence threshold, choice of threshold value would have severely impact on the barcoding results. In this study, when we set the threshold value to 15, 16, or 17 bp (corresponding to *ca*. 1.89%, 2.02%, or 2.14% of mean length of the sequences, respectively), the 11 clades were successfully recognized as 11 MOTU, which is consistent with the delimitations of BFD and BPP. However, when the threshold value was set to 42–60 bp, only three MOTU were recognized, which coincides with the result of ABGD. Thus, methods of fixed threshold may obtain a resolution similar to that of BFD and BPP, but they severely rely on subjective choice of the threshold and lack strong statistical support.

## Conclusions

Based on the molecular and available morphological evidences, we consider that species diversity in the *K. odontotarsus* species group was underestimated and six unnamed lineages may exist in this group. *Kurixalus hainanus* is valid and *K. verrucosus* from Tibet actually refers to *K. naso*. Our empirical data indicated that coalescent-based Bayes factor delimitation (BFD) and Bayesian species delimitation (BPP) are more powerful than distance-based ABGD for delimiting less divergent species in that the ABGD method is obviously prone to lump less divergent clades in a same candidate species (false negatives). Although distance-based method of fixed threshold (jMOTU) may obtain a resolution similar to that of BFD and BPP, it severely relies on subjective choice of the threshold and lacks strong statistical support. Our results also showed that it is not certain that the priors for ancestral population size and root age have impact on BPP delimitation.

## Methods

### Sampling

All methods were carried out in accordance with relevant guidelines and regulations and all experimental protocols were approved by the Ethics Committee of Kunming Institute of Zoology, Chinese Academy of Sciences (no. SYDW–2013017). A total of 160 individuals of *K. odontotarsus* species group collected from 53 sites across China, Laos, Vietnam, Cambodia, Myanmar, and Thailand (Fig. 1) were included in this study and were tentatively categorized into four species (*K. odontotarsus*, *K. verrucosus*, *K. bisacculus*, *K. baliogaster*) based on Yu *et al*.^29^ (Supplementary Table S2). Of the 160 individuals, 124 specimens were sequenced by this study and homologous sequences of other 36 individuals were obtained from previous studies^32,33,50,54^. *Kurixalus appendiculatus*, *Kurixalus eiffingeri*, *Kurixalus idiootocus*, *Kurixalus banaensis*, *Kurixalus viridescens*, and *Kurixalus motokawai* were selected as hierarchical outgroups for phylogenetic analyses based on previous studies^34,40^.

### DNA extraction, PCR amplification, and sequencing

Genomic DNA was extracted from liver or muscle tissue fixed in 99% ethanol. Tissue samples were digested using proteinase K, and subsequently purified following a standard phenol/chloroform isolation and ethanol precipitation. Fragments of three mitochondrial genes (12S rRNA, 16S rRNA, and COI) and three nuclear genes (tyrosinase exon 1 [Tyr], Rag-1, and brain-derived neurotrophic factor [BDNF]) were amplified and sequenced. The primers used for amplification and sequencing were listed in Supplementary Table S3. PCR amplifications were performed in 50 µl reactions using the following cycling conditions: an initial denaturing step at 94 °C for 3 min; 35 cycles of denaturing at 94 °C for 60 s, annealing at 48–54 °C (48 °C for COI, 49 °C for Rag-1, 50 °C for 12S, 16S, and BDNF, and 54 °C for Tyr), and extending at 72 °C for 60s; and a final extending step of 72 °C for 10 min. Sequencing was performed directly using the corresponding PCR primers. DNA sequences of both strands were obtained using the BigDye Terminator v.3.1 on an ABI PRISM 3730 following the manufacturer’s instructions. All new sequences have been deposited in GenBank under Accession Nos. KX554415–KX554921 (Supplementary Table S2).

### Phylogenetic analysis

Sequences were aligned using CLUSTAL X v1.83^55^ with the default parameters and then the alignments were revised by eye. No hypervariable regions were found in alignments of either 12S or 16S rRNA genes. Nucleotide saturation was tested for mitochondrial genes by plotting numbers of transition and transversion against Kimura-2-parameter distance (K2P) using DAMBE v. 5.2.5^56^ and saturation plots were also examined separately for the first, second, and third positions of protein-coding COI sequences. Nuclear sequences containing more than one ambiguous site were resolved using PHASE 2.1.1^57^, for which the input files were prepared using SEQPHASE^58^.

We inferred the mtDNA gene trees using Bayesian inference method in MrBayes v3.1.2^59^. Three separated datasets were prepared for this analysis owing to the absence of homologous 12S plus 16S rRNA or COI sequences for some individuals on GenBank, one is comprised of COI sequences (dataset I), one is comprised of 12S rRNA and 16S rRNA sequences (dataset II), and one is the combination of COI and rRNA sequences with all taxa for which sequences of the three genes are available (dataset III). The dataset I was partitioned by codon positions and the dataset III was divided into five partitions by genes and codon positions. The best substitution model for each partition was selected based on the Akaike information criterion in ModelTest v3.7^60^. Two runs were performed simultaneously with four Markov chains starting from random trees, and the Markov chains were run for 5,000,000 generations and sampled every 100 generations. Convergence and burn-in were checked using the program Tracer v1.6^61^. The first 25% of the trees were discarded as burn-in and the remaining trees were used to construct a majority-rule consensus tree showing all compatible partitions (contype = allcompat) and to estimate Bayesian posterior probabilities.

Besides Bayesian inference, neighbor-joining (NJ) analysis was also conducted in MEGA v.5.0^62^ based on COI sequences and the nodal support was estimated using 1,000 bootstrap pseudoreplicates. Uncorrected p-distances between and within clades were calculated in MEGA v.5.0 and distribution of pairwise genetic distances (uncorrected p-distance) between individuals was produced for COI sequences.

We also constructed the network of nuDNA. Firstly neighbor-joining (NJ) tree was constructed for each nuclear gene based on the uncorrected p-distance in MEGA v.5.0 and then the sequence alignment and NJ tree were imported into Haploviewer^63^ to visualize the network of haplotypes.

### Coalescent-based Species delimitation

Two coalescent-based statistical methods were used to delimit species boundaries among *K. odontotarsus* species group based on the mtDNA. Firstly, coupled with Bayesian species tree inference in BEAST 1.8.0^64^ using the *BEAST option^65^, the Bayes factor delimitation (BFD) approach^17^ was implemented to evaluate the competing models that were formulated based on current taxonomy and the obtained mtDNA gene tree. For each model, individuals were assigned to different species. Substitution model was determined using ModelTest 3.7 and all analyses employed an uncorrelated lognormal relaxed molecular clock with the clock rate of mtDNA was fixed to 1.0. Standard runs were conducted for 20 million generations, assuming a Yule process of species tree prior and piecewise linear & constant root of population size model. After the standard MCMC chain has finished, marginal likelihood estimation (MLE) was performed using both path sampling (PS)^30^ and stepping-stone (SS)^31^ via an additional run of four million generations of 100 path-steps (400 million generations). To get values of effective sample size (ESS) for parameters of MLE higher than 200 and to increase the accuracy of path sampling and stepping-stone sampling, the analysis was repeated four times for each delimitation model in BEAST 1.8.0 through the CRPRES Science Gateway^66^, and the final marginal likelihoods were estimated based on the combination of the four runs. Then, Bayes factors between alternative delimitation models were calculated by subtracting the MLE values for two models and multiplying the difference by two (BF = 2 × [model1 − model2]). The strength of support from BF comparisons was evaluated using the framework of Kass & Raftery^67^. The better model was chosen when the BF value was greater than 2. A value greater than 6 but lower than 10 is indicative of strong support and a value greater than 10 is decisive. For this method, the rRNA data and COI sequences were analyzed separately because *BEAST does not allow missing data and COI sequence is unavailable for some individuals, which will increase the difficulty in achieving convergence because there will be no information in the data concerning the placement of such a taxon in the genealogy^68^.

Secondly, unguided Bayesian species delimitation^69^ was conducted using the program Bayesian Phylogenetic and Phylogeography (BPP v3.2a)^70^ by allowing changes in the species tree topology (analysis A11). This method uses the multispecies coalescent model to compare different models of species delimitation and species phylogeny through reversible-jump Markov chain Monte Carlo (rjMCMC) analyses in a Bayesian framework. For this analysis, the rRNA and COI alignments were analyzed together as two loci and all the 160 individuals of *K. odontotarsus* species group were included because this method allows that different loci can have different numbers of sequences and some species can be missing at some loci. Considering the prior distributions on the ancestral population size (*θ*) and root age (τ) can affect the posterior probabilities of tested models^16^, we evaluated three different combinations of gamma distribution priors for *θ* and τ to confirm the stability of our results following Leaché & Fujita^44^. The first combination of priors assumed relatively large ancestral population sizes and deep divergences: *θ* ~ G (2, 50) and τ ~ G (2, 50). The second combination of priors assumed relatively small ancestral population sizes and shallow divergences among species: *θ* ~ G (2, 1000) and τ ~ G (2, 1000). The final combination is a mixture of priors that assume large ancestral populations sizes *θ* ~ G (2, 50) and relatively shallow divergences among species τ ~ G (2, 1000), which is a conservative combination of priors that should favour models containing fewer species^44^ (but see^42^). We conducted analyses under both rjMCMC algorithm 0 (*ɛ* = 2) and algorithm 1 (*α* = 2, *m* = 1), and each analysis was run twice using different random number seeds to confirm that the results are stable across runs. The rjMCMC was run for 200,000 generations and sampled every 2 generation with a burn-in period of 40,000 generations.

### Distance-based DNA barcoding

Two distance-based methods of DNA barcoding were performed. Firstly, we used the Automated Barcode Gap Discovery method (ABGD)^22^, a distance-based barcoding method that was widely used at the present (e.g.,^71–73^), to detect potential barcode gap and to partition the data into different groups for COI sequences. This analysis was performed on a web interface (wwwabi.snv.jussieu.fr/public/abgd/) using the simple distance model (p-distance) and a gap width of 1.5. The prior for the maximum value of intraspecific divergence (*P*) was set to ranges from 0.001 to 0.1 and the number of recursive steps is the default (10). Here we did not use the K2P model because use of this model for barcoding is questionable^74–76^ and it is not appropriate for closely related sequences^75^; instead uncorrected p-distances should be used^74,75,77^.

Secondly, a method of fixed threshold was performed to cluster the COI sequences into molecular operational taxonomic units (MOTU) in jMOTU^23^. The value of low blast identity filter was set to 97% according to the user guide and the value of minimum alignment length in base pairs was set to 658 bp because COI sequences obtained from GenBank overlap with our own sequences in 658 bp. For this analysis, a range of thresholds (from 1 to 60 bp) were tested.

## Electronic supplementary material

Supplementary Information
