# Supplementary material for: Coalescent-based delimitation outperforms distance-based methods for delineating less divergent species: the case of Kurixalus odontotarsus species group
Source: Sci Rep. 2017 Nov 23;7:16124. doi: 10.1038/s41598-017-16309-1 (PMC5700917; doi:10.1038/s41598-017-16309-1)
Supplement: Supplementary file 1 — Supplementary Information [file 41598_2017_16309_MOESM1_ESM.pdf]

Coalescent-based delimitation outperforms distance-based methods for delineating less divergent species: the case of *Kurixalus odontotarsus* species group

Guohua Yu[1,3], Dingqi Rao[1,3], Masafumi Matsui[2] & Junxing Yang*

[1]State Key Laboratory of Genetic Resources and Evolution, Kunming Institute of Zoology, Chinese Academy of Sciences, 32 Jiaochang Donglu, Kunming, Yunnan 650223, China. [2]Graduate School of Human and Environmental Studies, Kyoto University, Yoshida Nihonmatsu, Kakyo-ku, Kyoto 606-8501, Japan.

[3]These authors contributed equally to this work.

***Correspondence:** Junxing Yang, e-mail: yangjx@mail.kiz.ac.cn

Supplementary Tables

Table S1. Morphological comparison between different lineages.

Species	Male SVL (mm)	Snout	Vocal sac	Ventral surface	Dorsum	Serrated dermal fringes on limbs	Omosternum
Lineage A (*K. naso*)	29.6–34.3 (31.9 ± 1.41, n = 8)	pointed with a dermal projection	single	few small spots scattered; belly granular, chin and breast finely granular	rough	present	unknown
Lineage B (*K. sp*)	28.8–31.5 (n = 2)	pointed with a dermal projection	single	small darker spots; belly granular, chin and breast finely granular	rough	present	unknown
Lineage C (*K. sp*)	31.7–34.6 (32.7 ± 1.12, n = 11)	pointed with a dermal projection	single	few spots scattered on the belly; belly granular, chin and breast finely granular	rough	present	unknown
Lineage D (*K. odontotarsus*)	32.1–34.3 (33.3 ± 0.91, n = 8)	pointed with a dermal projection	single	lot of spots scattered on the belly; belly granular, chin and breast finely granular	rough	present	not forked
Lineage E (*K. bisacculus*)	30–31.5 (n = 3)	pointed with a dermal projection	two	chin clouded brownish; belly granular, chin and breast finely granular	rough	present	unknown
Lineage F (*K. sp*)	32.7–34.1 (33.3 ± 0.55, n = 8)	pointed with a dermal projection	single	lot of spots scattered on chin, chest, and belly; belly granular, chin and breast finely granular	rough	present	unknown
Lineage G (*K. sp*)	31 (n = 1)	pointed with a dermal projection	single	black spots scattered on chin and breast, few spots on the belly; belly, chin, and breast granular	rough	present	unknown
Lineage H (*K. sp*)	27.0–30.6 (28.5 ± 1.3, n = 7)	pointed with a dermal projection	single	chin clouded brownish, few or no spots on the belly; belly granular, chin and breast finely granular	rough	present	unknown

Lineage I (*K. baliogaster*)	33.0–33.3 (n = 2)	pointed with a dermal projection	single	lot of big spots scattered on chin, breast, and belly; belly granular, chin and breast finely granular	smooth	absent	unknown
Liineage J (*K. hainanus*)	32.4–34.7 (33.6 ± 0.77, n = 9)	pointed with a dermal projection	single	lot of spots scattered on chin, breast, and belly; belly granular, chin and breast finely granular	rough	present	forked
Lineage K (*K. sp*)	No data	pointed with a dermal projection	unknown	spots scattered on chin but few spots on chest and belly; chin and breast granular	rough	present	unknown
K. verrucosus	No data	Rounded, no dermal projection	unknown	Lot of spots scattered on chin, breast, and belly; belly granular, chin and breast smooth	rough	present	unknown

Table S2. Samples used in this study.

Species	Locality	Locality ID	Voucher No	12S	16S	COI	TYR	RAG1	BNDF
K. appendiculatus	Bukit Sarang, Sarawak, Malaysia	–	FMNH 267896	JQ060948	JQ060937	KX554539	–	–	–
K. eiffingeri		–	–	AB933305	AB933305	DQ468681	–	–	–
K. idiootocus		–	–	AB933306	AB933306	DQ468682			–
K. banaensis	Krong Pa, Gia Lai, Vietnam	–	ROM 32986	GQ285667	GQ285667	–	–	–	–
K. viridescens	Hon Ba, Khanh Hoa, Vietnam	–	VNMN 03802	AB933284	AB933284	–	–	–	–
K. motokawai	Kon Tum, Vietnam	–	VNMN 03458	LC002888	LC002888	–	–	–	–
K. verrucosus	Muotuo, Tibet, China	1	Rao 06193	KX554415	KX554477	KX554540	KX554649	KX554741	KX554854
	Muotuo, Tibet, China	1	Rao 06194	KX554416	KX554478	KX554541	KX554650	KX554742	–
	Muotuo, Tibet, China	1	Rao 06195	KX554417	KX554479	KX554542	–	–	–
	Muotuo, Tibet, China	1	Rao 06196	KX554418	KX554480	KX554543	–	–	–
	Muotuo, Tibet, China	1	Rao 06201	KX554419	KX554481	KX554544	KX554651	KX554743	KX554855
	Muotuo, Tibet, China	1	Rao 06202	KX554420	KX554482	KX554545	KX554652	KX554744	KX554856
	Muotuo, Tibet, China	1	Rao 06203	KX554421	KX554483	KX554546	–	–	–
	Muotuo, Tibet, China	1	Rao 06301	KX554422	KX554484	KX554547	KX554653	KX554745	–
	Muotuo, Tibet, China	1	Rao 06302	KX554423	KX554485	KX554548	KX554654	KX554746	–
	Muotuo, Tibet, China	1	Rao 06303	KX554424	KX554486	KX554549	–	–	–
	Muotuo, Tibet, China	1	Rao 06304	KX554425	KX554487	KX554550	–	–	–
	Muotuo, Tibet, China	1	Rao 06305	KX554426	KX554488	KX554551	KX554655	KX554747	KX554857
	Muotuo, Tibet, China	1	Rao 06306	KX554427	KX554489	–	KX554656	KX554748	–
	Muotuo, Tibet, China	1	Rao 06308	KX554428	KX554490	–	KX554657	KX554749	–
	Nagmung, Kachin, Myanmar	2	CAS 224381	GU227274	GU227329	KX554552	JQ060917	KX554750	–
	Nagmung, Kachin, Myanmar	2	CAS 224563	GU227275	GU227330	KX554553	KX554658	KX554751	KX554858
	Nagmung, Kachin, Myanmar	2	CAS 225128	GU227276	GU227331	KX554554	JQ060918	KX554752	KX554859
	Tai Nai, Kachin, Myanmar	3	CAS 231489	GU227277	GU227332	KX554555	KX554659	KX554753	KX554860

Table S2 (*continued*)

Species	Locality	Locality ID	Voucher No	12S	16S	COI	TYR	RAG1	BNDF
	Mohynin, Kachin, Myanmar	4	CAS 231491	GU227278	GU227333	KX554556	JQ060919	KX554754	KX554861
	Longdao, Ruili, Yunnan, China	5	Rao 14102901	KX554429	KX554491	KX554557	KX554660	KX554755	–
	Longdao, Ruili, Yunnan, China	5	Rao 14102902	KX554430	KX554492	KX554558	KX554661	KX554756	–
	Longdao, Ruili, Yunnan, China	5	Rao 14102903	KX554431	KX554493	KX554559	KX554662	KX554757	–
	Nanjingli, Ruili, Yunnan, China	6	Rao 14102904	KX554432	KX554494	KX554560	KX554663	KX554758	–
	Nanjingli, Ruili, Yunnan, China	6	Rao 14102905	KX554433	KX554495	KX554561	KX554664	KX554759	KX554862
	Nanjingli, Ruili, Yunnan, China	6	Rao 14102906	KX554434	KX554496	KX554562	KX554665	KX554760	–
	Yingjiang, Yunnan, China	7	Rao 14102908	KX554435	KX554497	KX554563	KX554666	KX554761	KX554863
	Yingjiang, Yunnan, China	7	Rao 14102909	KX554436	KX554498	KX554564	KX554667	KX554762	–
	Yingjiang, Yunnan, China	7	Rao 14102910	KX554437	KX554499	KX554565	KX554668	KX554763	–
	Yingjiang, Yunnan, China	7	Rao 14102911	KX554438	KX554500	KX554566	KX554669	KX554764	–
	Yingjiang, Yunnan, China	7	Rao 14102912	KX554439	KX554501	KX554567	KX554670	KX554765	–
	Yingjiang, Yunnan, China	7	Rao 14102913	KX554440	KX554502	KX554568	KX554671	KX554766	–
K. odontotarsus	Cangyuan, Yunnan, China	8	Rao 14001643	KX554441	KX554503	KX554569	KX554672	KX554767	KX554864
	Cangyuan, Yunnan, China	8	Rao 14102907	KX554442	KX554504	KX554570	KX554673	KX554768	KX554865
	Bada, Yunnan, China	9	Rao 14111307	KX554443	KX554505	KX554571	KX554674	KX554769	KX554866
	Mengyang, Yunnan, China	10	YGH 090174	GU227232	GU227281	KX554572	–	KX554770	–
	Mengyang, Yunnan, China	10	YGH 090175	GU227233	GU227282	KX554573	KX554675	KX554771	–
	Mengyang, Yunnan, China	10	YGH 090176	GU227234	GU227283	KX554574	KX554676	KX554772	–
	Mengyang, Yunnan, China	10	YGH 090177	GU227235	GU227284	–	KX554677	KX554773	KX554867
	Mengyang, Yunnan, China	10	YGH 090178	KX554444	KX554506	–	–	KX554774	–
	Mengyang, Yunnan, China	10	YGH 090179	GU227236	GU227285	–	KX554678	KX554775	KX554868
	Dadugang, Yunnan, China	11	YGH 71170	GU227237	GU227286	–	–	–	–
	Dadugang, Yunnan, China	11	YGH 71171	GU227238	GU227287	–	–	–	–

Table S2 (*continued*)

Species	Locality	Locality ID	Voucher No	12S	16S	COI	TYR	RAG1	BNDF
	Menglun, Yunnan, China	12	KIZ 060821122	EF564456	GU227288	–	KX554679	KX554776	–
	Menglun, Yunnan, China	12	Rao 14111401	KX554445	KX554507	KX554575	KX554680	KX554777	KX554869
	Caiyanghe, Yunnan, China	13	YGH 090130	GU227239	GU227289	–	KX554681	KX554778	–
	Caiyanghe, Yunnan, China	13	YGH 090131	GU227240	GU227290	KX554576	KX554682	KX554779	KX554870
	Caiyanghe, Yunnan, China	13	YGH 090132	GU227241	GU227291	–	KX554683	KX554780	KX554871
	Caiyanghe, Yunnan, China	13	YGH 090133	GU227242	GU227292	KX554577	KX554684	KX554781	KX554872
	Caiyanghe, Yunnan, China	13	YGH 090134	GU227243	GU227293	–	KX554685	KX554782	KX554873
	Caiyanghe, Yunnan, China	13	KIZ 060821030	EF564455	GU227294	KX554578	EU924566	KX554783	–
	Nathen, Phongsali, Laos	14	MNHN 2004.0351	–	–	KR087747	–	–	–
	Long Nai Khao, Phongsali, Laos	15	MNHN 2004.0352	–	–	KR087748	–	–	–
	Phoufa Mountain, Phongsali, Laos	16	MNHN 2004.0353	–	–	KR087749	–	–	–
	Ban Keng Koung, Louangphrabang, Laos	17	MNHN 2006.2456	–	–	KR087750	–	–	–
	Luang Namtha, Laos	18	FMNH 271334	KC465815	KC465815	–	–	–	–
	Sa Pa, Lao Cai, Vietnam	19	ROM 38436	KC465817	KC465817	–	–	–	–
	Sa Pa, Lao Cai, Vietnam	19	ROM 38955	KC465818	KC465818	–	–	–	–
K. bisacculus	Sa pa, Lao Cai, Vietnam	19	MNHN 1999.5941	–	–	KR087741	–	–	–
	Sa pa, Lao Cai, Vietnam	19	MNHN 1999.5942	–	–	KR087742	–	–	–
	Sa Pa, Lao Cai, Vietnam	19	ROM 38943	KC465797	KC465797	–	–	–	–
	Sa Pa, Lao Cai, Vietnam	19	ROM 38962	KC465799	KC465799	–	–	–	–
	Sa Pa, Lao Cai, Vietnam	19	ROM 38981	KC465800	KC465800	–	–	–	–
	Mengzi, Yunnan, China	20	1506227	KX554446	KX554508	KX554579	–	–	–
	Mengzi, Yunnan, China	20	1506228	KX554447	KX554509	KX554580	–	–	–
	Mengzi, Yunnan, China	20	1506229	KX554448	KX554510	KX554581	–	–	–
	Pingbian, Yunnan, China	21	YGH 080166	GU227244	GU227295	KX554582	KX554686	KX554784	–

Table S2 (*continued*)

Species	Locality	Locality ID	Voucher No	12S	16S	COI	TYR	RAG1	BNDF
	Pingbian, Yunnan, China	21	YGH 080168	GU227245	GU227296	KX554583	KX554687	KX554785	–
	Pingbian, Yunnan, China	21	YGH 080169	GU227246	GU227297	KX554584	KX554688	KX554786	KX554874
	Pingbian, Yunnan, China	21	YGH 080170	GU227247	GU227298	KX554585	KX554689	KX554787	KX554875
	Jinping, Yunnan, China	22	KIZ 060821124	KX554449	KX554511	–	KX554690	–	–
	Thanh Hoa, Vietnam	23	VNMN 03806	AB933292	AB933292	–	–	–	–
	Thanh Hoa, Vietnam	23	VNMN 03807	AB933293	AB933293	–	–	–	–
	Thanh Hoa, Vietnam	23	VNMN 03808	AB933294	AB933294	–	–	–	–
	Thanh Hoa, Vietnam	23	VNMN 03809	AB933295	AB933295	–	–	–	–
	Thanh Hoa, Vietnam	23	VNMN 03810	AB933296	AB933296	–	–	–	–
	Tuong Duong Dist, Nghe An, Vietnam	24	FMNH 255661	KX554450	KX554512	KX554586	KX554691	KX554788	KX554876
	Con Cuong Dist, Nghe An, Vietnam	25	FMNH 255654	KX554451	KX554513	KX554587	KX554692	KX554789	–
	Con Cuong Dist, Nghe An, Vietnam	25	FMNH 255655	KX554452	KX554514	KX554588	KX554693	KX554790	KX554877
	Con Cuong Dist, Nghe An, Vietnam	25	FMNH 255656	KX554453	KX554515	KX554589	KX554694	KX554791	KX554878
	Con Cuong Dist, Nghe An, Vietnam	25	FMNH 255657	KX554454	KX554516	KX554590	KX554695	KX554792	–
	Con Cuong Dist, Nghe An, Vietnam	25	ROM 27969	KC465798	KC465798	–	–	–	–
	Nakai Dist, Khammouan, Lao	26	FMNH 256452	KX554455	KX554517	KX554591	KX554696	KX554793	–
	Nakai Dist, Khammouan, Lao	26	FMNH 256453	KX554456	KX554518	KX554592	KX554697	KX554794	KX554879
	Nakai Dist, Khammouan, Lao	26	FMNH 256454	KX554457	KX554519	KX554593	KX554698	KX554795	KX554880
	Pakxong Dist, Champasak, Lao	27	FMNH 257903	KX554458	KX554520	KX554594	KX554699	KX554796	KX554881
	Wenshan, Yunnan, China	28	KIZ 3315	EF564459	GU227305	KX554595	KX554700	KX554797	–
	Wenshan, Yunnan, China	28	KIZ 3317	EF564460	GU227306	KX554596	KX554701	KX554798	KX554882
	Wenshan, Yunnan, China	28	YGH 71153	GU227251	GU227302	KX554597	KX554702	KX554799	KX554883
	Wenshan, Yunnan, China	28	YGH 71154	GU227252	GU227303	KX554598	KX554703	KX554800	–
	Wenshan, Yunnan, China	28	YGH 090044	GU227248	GU227299	KX554599	KX554704	KX554801	–

Table S2 (*continued*)

Species	Locality	Locality ID	Voucher No	12S	16S	COI	TYR	RAG1	BNDF
	Wenshan, Yunnan, China	28	YGH 090046	GU227249	GU227300	KX554600	KX554705	KX554802	KX554884
	Wenshan, Yunnan, China	28	YGH 71152	GU227250	GU227301	KX554601	KX554706	KX554803	KX554885
	Wenshan, Yunnan, China	28	KIZ 0602003	EF564458	GU227304	KX554602	KX554707	KX554804	KX554886
	Wenshan, Yunnan, China	28	Rao 14111306	KX554459	KX554521	KX554603	KX554708	KX554805	KX554887
	Jingxi, Guangxi, China	29	YGH 090280	GU227259	GU227313	KX554604	KX554709	KX554806	KX554888
	Jingxi, Guangxi, China	29	YGH 090281	GU227260	GU227314	KX554605	KX554710	KX554807	–
	Jingxi, Guangxi, China	29	YGH 090282	GU227261	GU227315	KX554606	KX554711	KX554808	KX554889
	Jingxi, Guangxi, China	29	YGH 090283	GU227262	GU227316	KX554607	–	–	–
	Jingxi, Guangxi, China	29	YGH 090284	GU227263	GU227317	KX554608	KX554712	KX554809	KX554890
	Jingxi, Guangxi, China	29	YGH 090285	KX554460	KX554522	–	KX554713	KX554810	–
	Jingxi, Guangxi, China	29	YGH 090286	GU227264	GU227318	–	–	KX554811	–
	Libo, Guizhou, China	30	YGH 090081	GU227253	GU227307	KX554609	KX554714	KX554812	KX554891
	Nanning, Guangxi, China	31	YGH 090266	GU227254	GU227308	KX554610	–	KX554813	–
	Nanning, Guangxi, China	31	YGH 090267	GU227255	GU227309	KX554611	KX554715	KX554814	–
	Nanning, Guangxi, China	31	YGH 090268	GU227256	GU227310	KX554612	KX554716	KX554815	KX554892
	Nanning, Guangxi, China	31	YGH 090269	GU227257	GU227311	KX554613	–	KX554816	–
	Nanning, Guangxi, China	31	YGH 090270	GU227258	GU227312	KX554614	KX554717	KX554817	KX554893
	Jinxiu, Guangxi, China	32	KIZ 060821015	EF564454	GU227319	KX554615	JQ060914	–	–
	Longmeng, Guangdong, China	33	YGH 090201	GU227265	GU227320	KX554616	KX554718	KX554818	KX554894
	Longmeng, Guangdong, China	33	YGH 090202	GU227266	GU227321	KX554617	KX554719	KX554819	KX554895
	Longmeng, Guangdong, China	33	YGH 090203	GU227267	GU227322	–	–	KX554820	–
	Longmeng, Guangdong, China	33	YGH 090204	GU227268	GU227323	KX554618	KX554720	KX554821	KX554896
	Longmeng, Guangdong, China	33	YGH 090205	GU227269	GU227324	KX554619	KX554721	KX554822	KX554897
	Longmeng, Guangdong, China	33	YGH 090206	GU227270	GU227325	–	–	KX554823	–

Table S2 (*continued*)

Species	Locality	Locality ID	Voucher No	12S	16S	COI	TYR	RAG1	BNDF
	Longmeng, Guangdong, China	33	YGH 090207	GU227271	GU227326	–	–	KX554824	–
	Diaoluo Mts, Hainan, China	34	YGH 080078	GU227273	GU227328	–	–	–	–
	Diaoluo Mts, Hainan, China	34	HNNU A1180	EU215548	EU215548	–	–	GQ285749	–
	Diaoluo Mts, Hainan, China	34	Rao14111301	KX554461	KX554523	KX554620	KX554722	KX554825	KX554898
	Diaoluo Mts, Hainan, China	34	Rao14111302	KX554462	KX554524	KX554621	KX554723	KX554826	KX554899
	Diaoluo Mts, Hainan, China	34	Rao14111303	KX554463	KX554525	KX554622	KX554724	KX554827	KX554900
	Diaoluo Mts, Hainan, China	34	Rao14111304	KX554464	KX554526	KX554623	KX554725	KX554828	KX554901
	Mt. Wuzhi, Hainan, China	35	MVZ Herp 236722	JQ060939	JQ060928	KX554624	JQ060915	KX554829	KX554902
	Bawangling, Hainan, China	36	MVZ Herp 236725	JQ060940	JQ060929	KX554625	JQ060916	KX554830	KX554903
	Ha Giang, Vietnam	37	VNMN 01561	AB933287	AB933287	–	–	–	–
	Pac Ban, Tuyen Quang, Vietnam	38	ROM 30042	KC465809	KC465809	–	–	–	–
	Cao Bang, Vietnam	39	ROM 36726	KC465802	KC465802	–	–	–	–
	Cao Bang, Vietnam	39	VNMN 03805	AB933288	AB933288	–	–	–	–
	Tam Dao, Vinh Phu, Vietnam	40	MVZ Herp 223856	JQ060941	JQ060930	KX554626	JQ060920	KX554831	–
	Tam Dao, Vinh Phu, Vietnam	40	MVZ Herp 223857	JQ060942	JQ060931	KX554627	KX554726	KX554832	KX554904
	Tam Dao, Vinh Phu, Vietnam	40	MVZ Herp 226463	JQ060943	JQ060932	KX554628	JQ060921	KX554833	KX554905
	Tam Dao, Vinh Phu, Vietnam	40	MVZ Herp 226464	JQ060944	JQ060933	KX554629	JQ060922	KX554834	KX554906
	Tam Dao, Vinh Phu, Vietnam	40	MVZ Herp 226465	JQ060945	JQ060934	KX554630	JQ060923	KX554835	–
	Tam Dao, Vinh Phu, Vietnam	40	MVZ Herp 226467	JQ060946	JQ060935	KX554631	JQ060924	KX554836	–
	Tam Dao, Vinh Phu, Vietnam	40	MVZ Herp 226468	JQ060947	JQ060936	KX554632	JQ060925	KX554837	–
	Tam Dao, Vinh Phu, Vietnam	40	K719	–	–	KR087739	–	–	–
	Tam Dao, Vinh Phu, Vietnam	40	2000.2944	–	–	KR087744	–	–	–
	Huu Lien, Lang So’n, Vietnam	41	2007.6281	–	–	KR087743	–	–	–
	Huu Lien, Lang So’n, Vietnam	41	2007.6276	–	–	KR087745	–	–	–

Table S2 (*continued*)

Species	Locality	Locality ID	Voucher No	12S	16S	COI	TYR	RAG1	BNDF
	Huu Lien, Lang So'n, Vietnam	41	2007.6277	–	–	KR087746	–	–	–
	Chi Linh, Hai Duong, Vietnam	42	ROM 36829	KC465812	KC465812	–	–	–	–
	Chi Linh, Hai Duong, Vietnam	42	ROM 36827	KC465813	KC465813	–	–	–	–
	Pua, Nan, Thailand	43	THNHM 10051	GU227279	GU227334	KX554633	JQ060912	KX554838	KX554907
	Pua, Nan, Thailand	43	THNHM 10052	GU227280	GU227335	KX554634	JQ060913	KX554839	KX554908
	Muang Sa Kaeo, Sa Kaeo, Thailand	44	FMNH 265820	KX554465	KX554527	KX554635	KX554727	KX554840	KX554909
	Phnom Samkos, Cambodia	45	LSUHC 9430	KX554466	KX554528	KX554636	KX554728	KX554841	KX554910
	Kampot Dist, Kampot Prov, Cambodia	46	FMNH 261898	KX554467	KX554529	KX554637	KX554729	KX554842	KX554911
	Kampot Dist, Kampot Prov, Cambodia	46	FMNH 261899	KX554468	KX554530	KX554638	KX554730	KX554843	KX554912
	Kampot Dist, Kampot Prov, Cambodia	46	FMNH 261900	KX554469	KX554531	KX554639	KX554731	KX554844	–
	Kampot Dist, Kampot Prov, Cambodia	46	FMNH 261901	KX554470	KX554532	KX554640	KX554732	KX554845	KX554913
	Kampot Dist, Kampot Prov, Cambodia	46	FMNH 261902	KX554471	KX554533	KX554641	KX554733	KX554846	KX554914
	Pilok, Kanchanaburi, Thailand	47	KUHE 35069	AB933291	AB933291	KX554642	KX554734	KX554847	KX554915
	Nakon Sri Tamarat, Thailand	48	KUHE 19428	AB933290	AB933290	KX554643	KX554735	KX554848	KX554916
	Phu Luanag, Loei, Thailand	49	KUHE 19330	KX554472	KX554534	KX554644	KX554736	KX554849	KX554917
	Phu Luanag, Loei, Thailand	49	KUHE 19333	KX554473	KX554535	KX554645	KX554737	KX554850	KX554918
K. baliogaster	Kon Tum, Vietnam	50	VNMN 03618	AB933300	AB933300	–	–	–	–
	Krong Pa, Gia Lai, Vietnam	51	Rom 33963	KX554474	KX554536	KX554646	KX554738	KX554851	KX554919
	Tram Lap, Gia Lai, Vietnam	52	Rom 29860	KX554475	KX554537	KX554647	KX554739	KX554852	KX554920
	Tram Lap, Gia Lai, Vietnam	52	Rom 29862	KX554476	KX554538	KX554648	KX554740	KX554853	KX554921
	Gia Lai, Vietnam	52	VNMN 03812	AB933298	AB933298	–	–	–	–
	Bi Doup, Lam Dong, Vietnam	53	KIZ 874	KC465821	KC465821	–	–	–	–

Table S3. Primers used for amplification and sequencing in this study.

Fragment	Primer name	Primer sequence	Product length (bp)	Source
12S	L1091	5’-AAA AAG CTT CAA ACT GGG ATT AGA TAC CCA CTA T-3’	401	1
	H1478	5’-TGA CTG CAG AGG GTG ACG GGC GGT GTG T-3’		1
16S	L2188	5’-AAA CTG GGC CTA AAA GCA GCC A-3’	876	2
	16H1	5’-CTC CGG TCT GAA CTC AGA TCA CGT AGG-3’		3
COI	Chmf4-M	5’-TYT CWA CWA AYC ATA AAG AYA TCG G-3’	817	Modified from Che *et al.*[4]
	H-tCOIc	5’-TGG TGG GCT CAT ACA ATA AAG C-3’		5
Tyr	Tyr1C	5’-GGC AGA GGA WCR TGC CAA GAT GT-3’	540	6
	Tyr1G	5’-TGC TGG GCR TCT CTC CAR TCC CA-3’		6
Rag-1	Rag1-KuF	5’-GAA TAC CCA GTG GAA GCG A-3’	926	Designed by this study
	Rag1-KuR	5’-TTT GAG TGC CTC TTG TCT CTC T-3’		Designed by this study
BDNF	BDNF-L	5’-ACC ATC CTT TTC CTK ACT ATG G-3’	701	7
	BDNF-R	5’-CTA TCT TCC CCT TTT AAT GGT C-3’		7

1. Kocher, T. D. *et al*. Dynamics of mitochondrial DNA evolution in animals: amplification and sequencing with conserved primers. *Proc. Natl. Acad. Sci. USA* **86**, 6196–6200 (1989).
2. Matsui, M. *et al*. Phylogenetic relationships of oriental torrent frogs in the genus *Amolops* and its allies (Amphibia, Anura, Ranidae). *Mol. Phylogenet. Evol.* **38**, 659–666 (2006).
3. Hedges, S. B. Molecular evidence for the origin of birds. *Proc. Natl. Acad. Sci. USA* **91**, 2621–2624 (1994).
4. Che, J. *et al*. Universal COI primers for DNA barcoding amphibians. *Mol. Ecol. Resour.* **12**, 247–258 (2012).
5. Stuart, B. L. & Parham, J. F. Molecular phylogeny of the critically endangered Indochinese box turtle (*Cuora galbinifrons*). *Mol. Phylogenet. Evol.* **31**, 164–177 (2004).
6. Bossuyt, F. & Milinkovitch, M. C. Convergent adaptive radiations in Madagascan and Asian ranid frogs reveal covariation between larval and adult traits. *Proc. Natl. Acad. Sci. USA* **97**, 6585–6590 (2000).
7. Van der Meijden, A. *et al*. Nuclear gene phylogeny of narrow-mouthed toads (Family: Microhylidae) and a discussion of competing hypotheses concerning their biogeographical origins. *Mol. Phylogenet. Evol.* **44**, 1017–1030 (2007).

Supplementary Figures

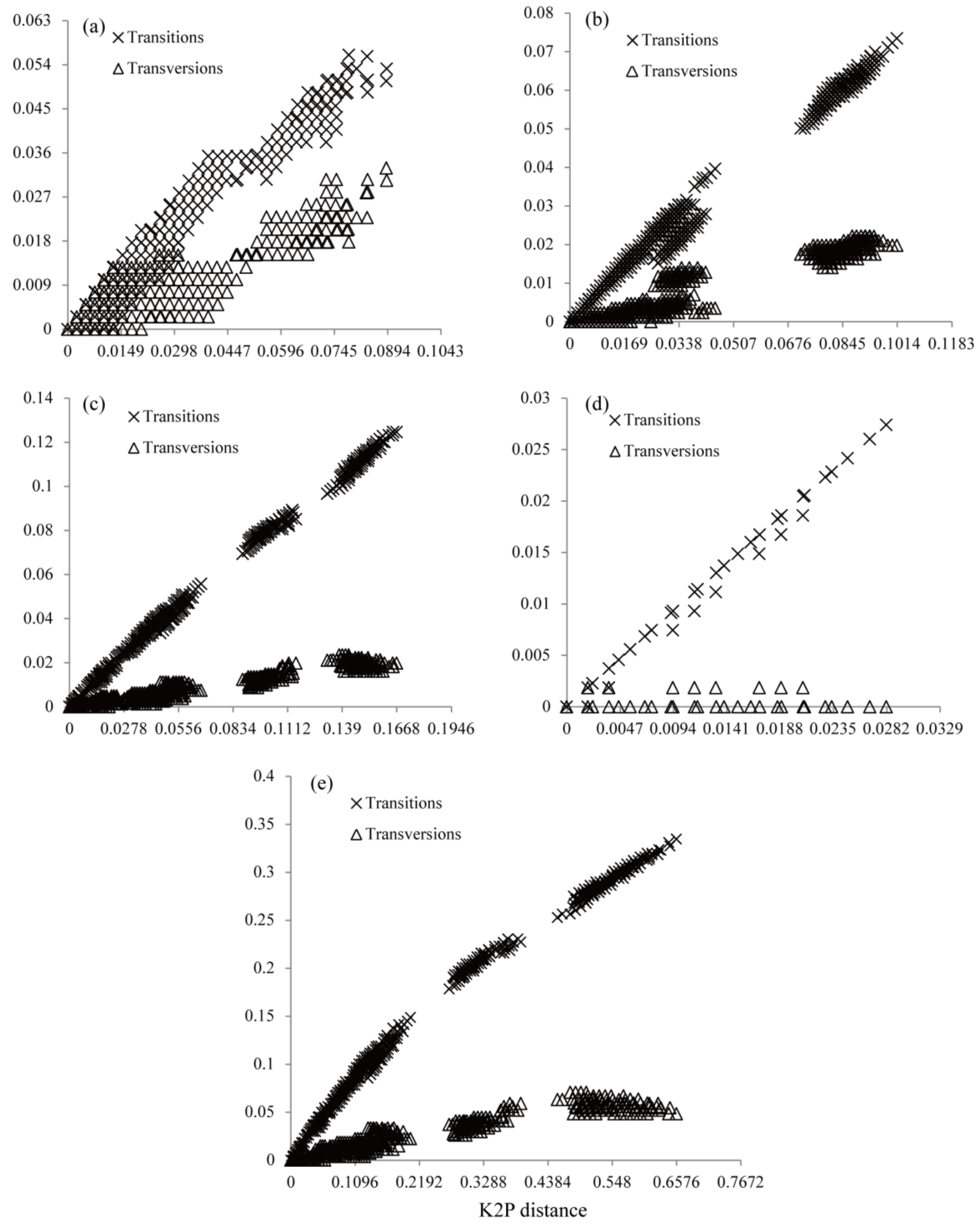

Figure S1. Transitions and transversions plotted against Kimura-2-parameter distance for 12S, 16S and COI sequences. (a) 12S; (b) 16S; (c) all nucleotides of COI; (d) codon positions 1 and 2 of COI; (e) codon position 3 of COI.

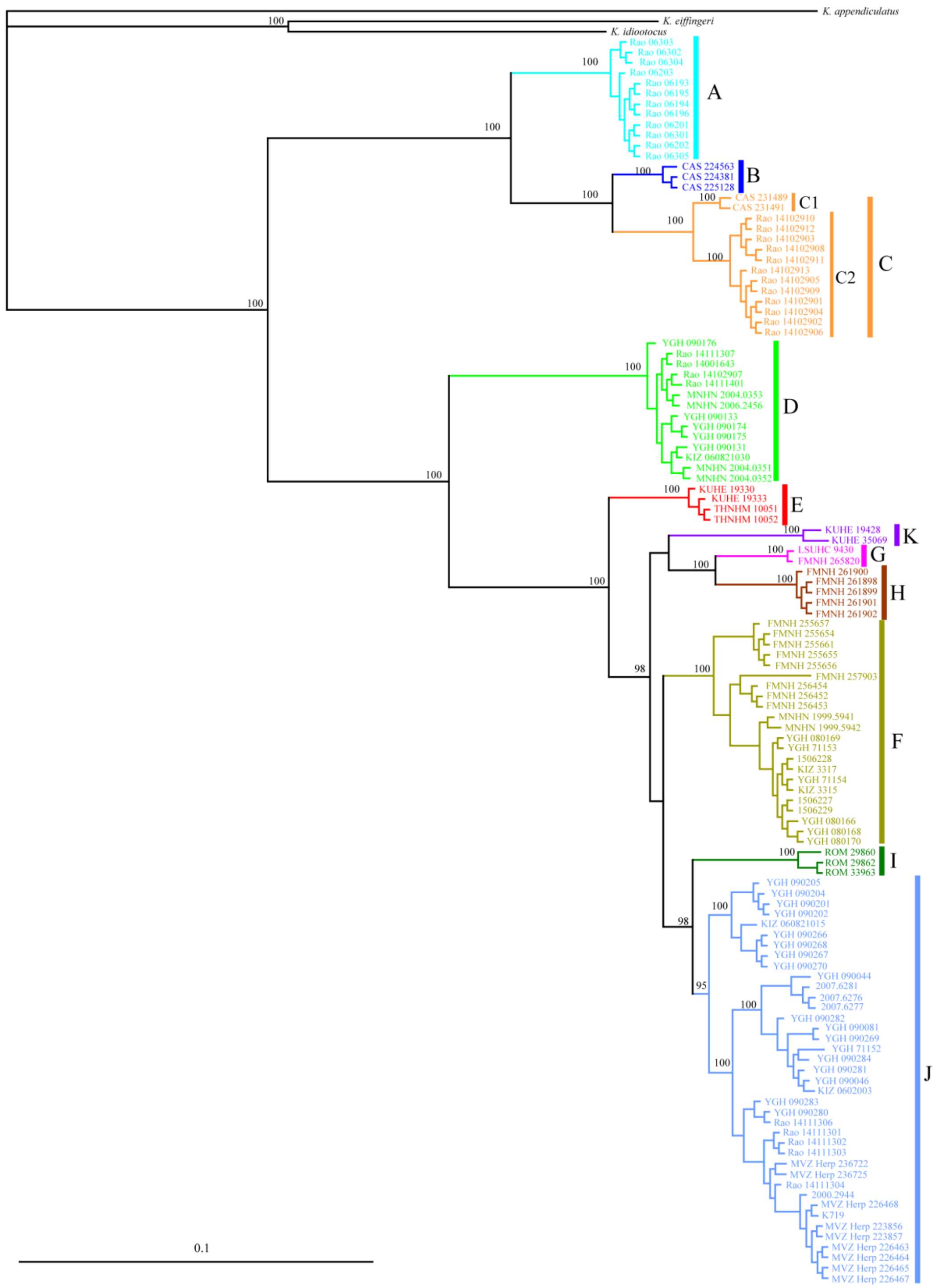

Figure S2. Bayesian dendrogram of the *K. odontotarsus* species group inferred from COI sequences. The nodal numbers are BPP values.

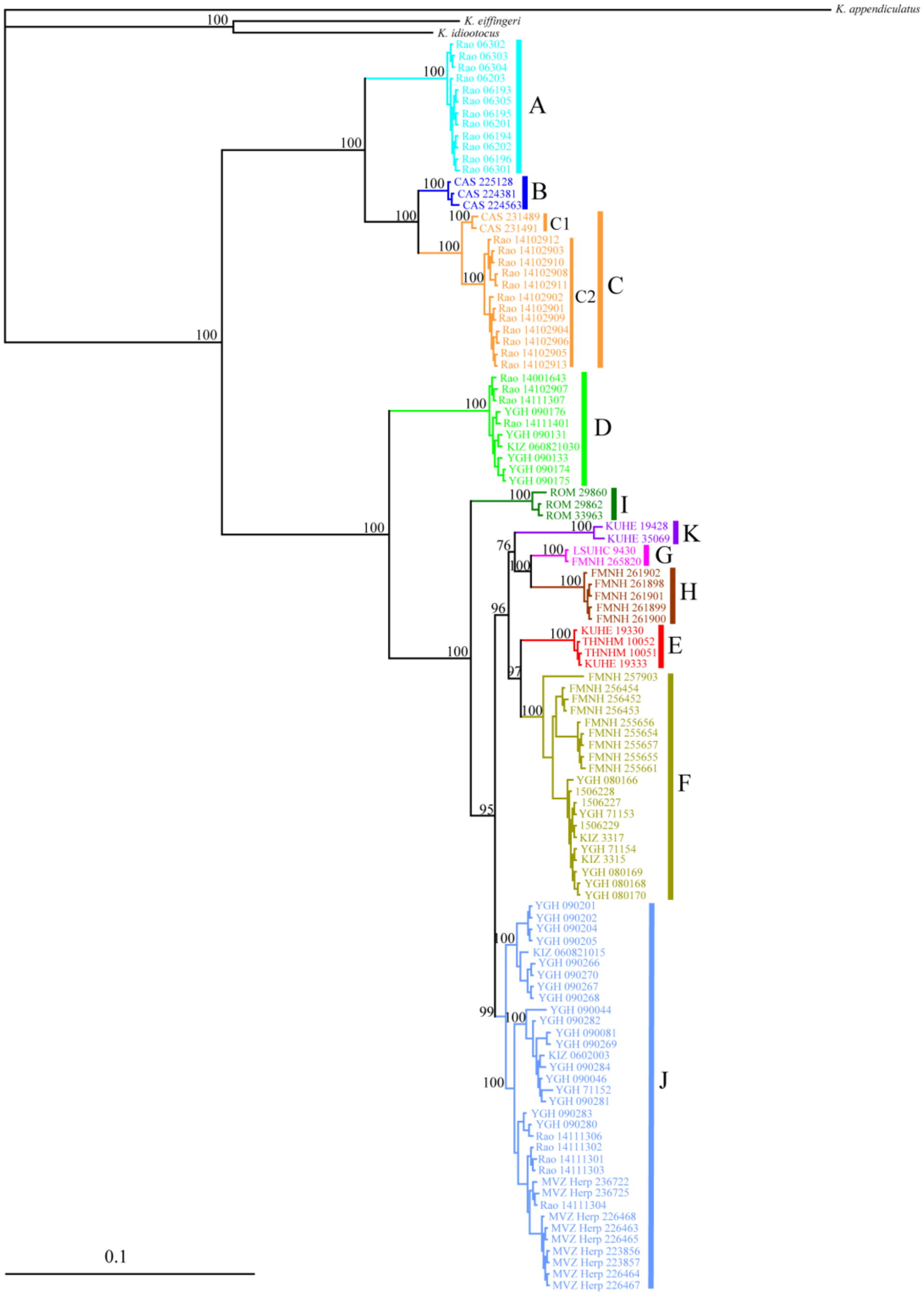

Figure S3. Bayesian dendrogram of the *K. odontotarsus* species group inferred from the combination of 12S, 16S and COI sequences. The nodal numbers are BPP values.

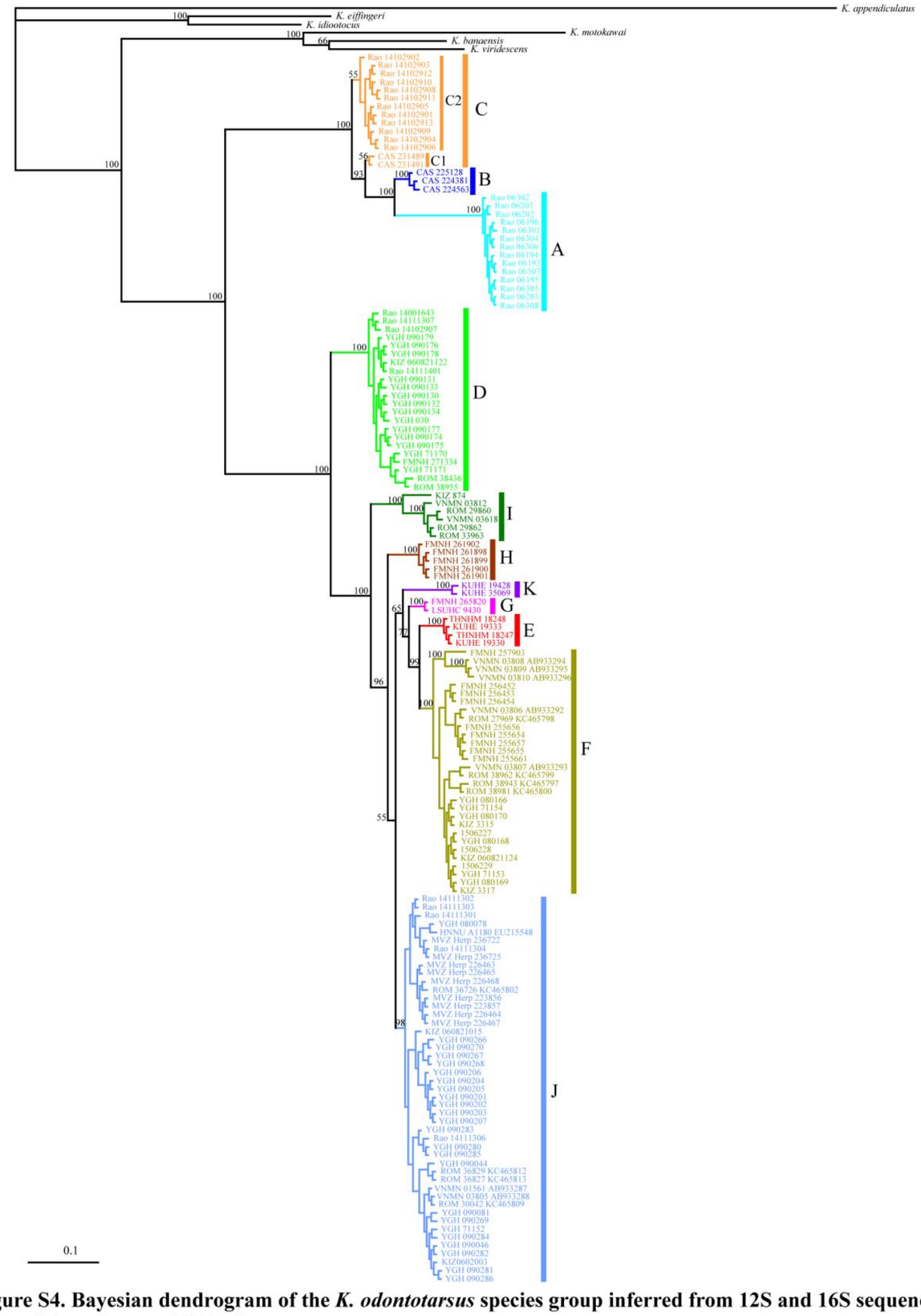

Figure S4. Bayesian dendrogram of the *K. odontotarsus* species group inferred from 12S and 16S sequences. The nodal numbers are BPP values.

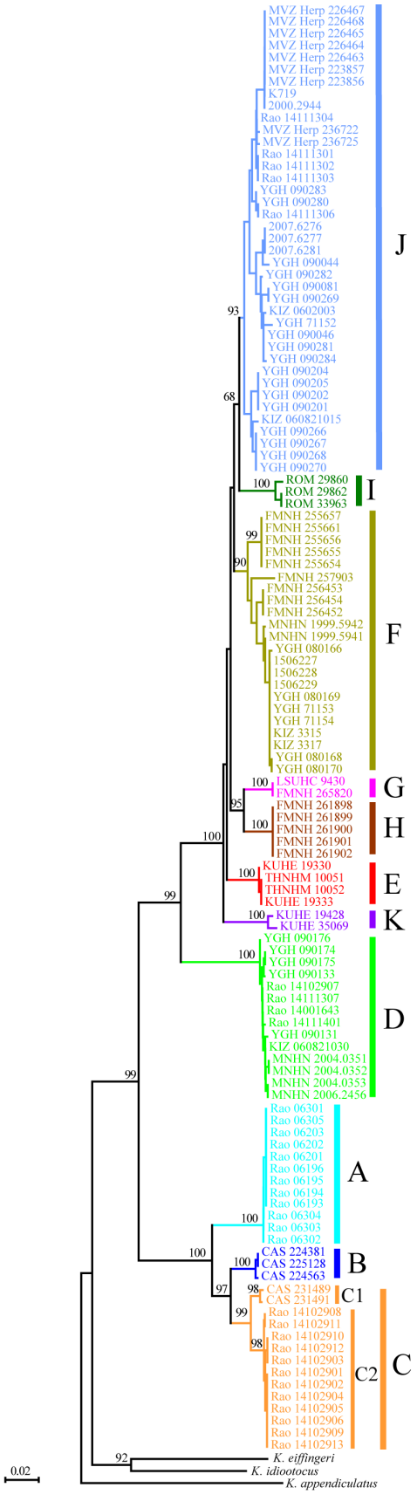

Figure S5. Neighbour-Joining (NJ) tree of the *K. odontotarsus* species group obtained from COI sequences. The nodal numbers are bootstrap values estimated by 1000 replicates.

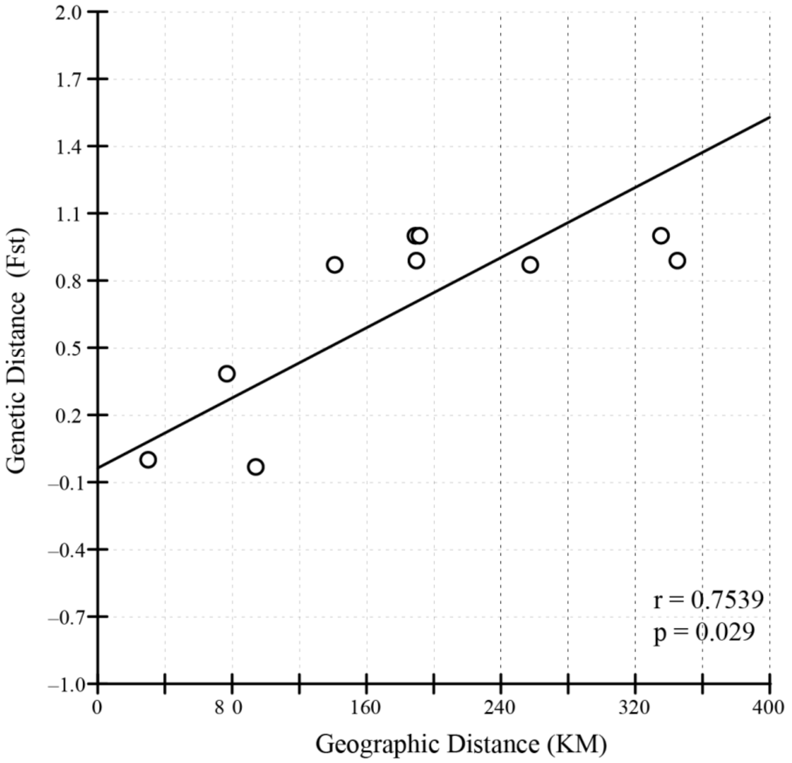

Figure S6. Correlation between genetic distance and graphical distance tested for clade C.
